# Clinical significance of genetic profiling based on different anatomic sites in patients with mucosal melanoma who received or did not receive immune checkpoint inhibitors

**DOI:** 10.1186/s12935-023-03032-3

**Published:** 2023-08-30

**Authors:** Hai-Yun Wang, Ye Liu, Ling Deng, Kuntai Jiang, Xin-Hua Yang, Xiao-Yan Wu, Kai-Hua Guo, Fang Wang

**Affiliations:** 1 Department of Pathology, Guangdong Provincial Clinical Research Center for Child Health, Guangzhou Women and Children’s Medical Center, Guangzhou Medical University, National Children’s Medical Center for South Central Region, 510623 Guangzhou, P. R. China; 2 Guangzhou Institute of Pediatrics, Guangdong Provincial Key Laboratory of Research in Structural Birth Defect Disease, Guangdong Provincial Clinical Research Center for Child Health, Guangzhou Women and Children’s Medical Center, Guangzhou Medical University, National Children’s Medical Center for South Central Region, 510623 Guangzhou, P. R. China; 3 Shenzhen Hospital, National Cancer Center, National Clinical Research Center for Cancer/Cancer Hospital, Chinese Academy of Medical Sciences and Peking Union Medical College, 518116 Shenzhen, China; 4 Department of Molecular Diagnostics, State Key Laboratory of Oncology in South China, Collaborative Innovation Center for Cancer Medicine, Sun Yat-Sen University Cancer Center, 510060 Guangzhou, P. R. China; 5 Department of Anatomy and Neurobiology, Zhongshan School of Medicine, Sun Yat-Sen University, 74 Zhongshan Road, 510080 Guangzhou, P. R. China

**Keywords:** Mucosal melanoma, Clinical significance, Primary site, Prognosis, Genetic alterations

## Abstract

**Background:**

To date, data on the efficacy of targeted therapies for mucosal melanoma (MM) are limited. In this study, we analyzed genetic alterations according to the primary site of origin, which could provide clues for targeted therapy for MM.

**Methods:**

We conducted a retrospective cohort study of 112 patients with MM. Targeted sequencing was performed to analyze genetic aberrations. Kaplan–Meier analysis was conducted with the log-rank test to compare the significance among subgroups.

**Results:**

In total, 112 patients with MM were included according to the anatomic sites: 38 (33.9%) in the head and neck, 22 (19.6%) in the genitourinary tract, 21 (18.8%) in the anorectum, 19 (17.0%) in the esophagus, 10 (8.9%) in the uvea, and 2 (1.8%) in the small bowel. The most significantly mutated genes included *BRAF (17%), KIT (15%), RAS (15%), TP53 (13%), NF1 (12%), SF3B1 (11%), GNA11 (7%), GNAQ (5%)*, and *FBXW7 (4%)*. A large number of chromosomal structural variants was found. The anatomic sites of esophagus and small bowel were independent risk factors for progression-free survival (PFS, hazard ratio [HR] 4.78, 95% confidence interval [CI] 2.42–9.45, P < 0.0001) and overall survival (OS, HR 5.26, 95% CI 2.51–11.03, P < 0.0001). Casitas B-lineage lymphoma (*CBL*) mutants showed significantly poorer PFS and OS. In contrast, MM patients who received immune checkpoint inhibitors (ICIs) had a significantly more favorable OS (HR 0.39, 95% CI 0.20–0.75, P = 0.008).

**Conclusions:**

Our findings reveal the genetic features of patients with MM, mainly across six anatomic sites, offering a potential avenue for targeted therapies.

**Supplementary Information:**

The online version contains supplementary material available at 10.1186/s12935-023-03032-3.

## Background

Primary mucosal melanomas (MM), arising from melanocytes in mucosal tissues lining the head and neck, respiratory, gastrointestinal, and urogenital tracts, account for 26% of all melanomas in Asian populations [1]. Atypical symptoms are commonly observed in the early stage because of the occult anatomic locations, and the median age at presentation is 70 years, which is later than that for cutaneous melanomas [2]. Moreover, the clinical presentation of MM is more aggressive and has a poorer prognosis, regardless of the stage at diagnosis, with a 5-year survival rate of 25% owing to the lack of effective targeted therapies [3].

Importantly, MM is markedly different from cutaneous melanoma in terms of biological aspects. Recently, whole-genome and exome sequencing have revealed the molecular landscape and potential oncogenic drivers of MM, which are characterized by greater copy number (CN) variations, structural variations (SVs), and a lower tumor mutation burden (TMB) [4]. Targeted therapy with *BRAF/MEK* tyrosine kinase inhibitors improves patient survival [5]. However, *BRAF* mutations are less frequent in MM and are susceptible to resistance, resulting in a lower effectivity of *BRAF/MEK* inhibitors [6]. Immune checkpoint inhibitors (ICIs) are also beneficial for MM, with a median progression-free survival (PFS) of only 3.9 months [7]. Although *BRAF, RAS, KIT, NF1*, and *SF3B1* have been identified as significantly mutated genes [4], there remains a lack of understanding and identification of oncogenic drivers in MM, probably owing to the rarity of samples and lack of preclinical models.

To further explore the molecular profiles of MM, we performed targeted next-generation sequencing (NGS) of 112 MM patients to explore the clinical significance of genetic characteristics potentially providing molecular targets and individualized treatments and provide a better mechanistic understanding of MM biology across the anatomic sites of MM.

## Methods

### Study participants

A total of 112 MM samples were collected from patients treated or consulted at the Sun Yat-sen University Cancer Center (SYSUCC, Guangzhou, China) between April 2007 and April 2022. Patients with cutaneous melanoma that had metastasized to the mucosa were excluded. The staging standard that we used followed the newest proposal for all anatomical sites published in April 2022 [8]. For all cases, the diagnoses were reviewed and confirmed by two experienced pathologists (Y. L. and F. W.). Clinical follow-up and treatment information as well as telephone interview data were available from the medical records of inpatients and outpatients (Supplementary Table S1). The study protocol was designed in accordance with the principles of the Declaration of Helsinki and approved by the Research Ethics Committee of the SYSUCC (No. B2016-069-01).

### DNA extraction

Formalin-fixed and paraffin-embedded (FFPE) tissue blocks were assessed using hematoxylin-eosin (H&E) staining, and regions containing minimum of 20% tumor cells in unstained sections were selected for microdissection and subsequent experiments. Genomic DNA was extracted from tumors and patient-matched normal tissues or white blood cells using a QIAamp DNA FFPE Tissue Kit (Qiagen, Hilden, Germany) according to the manufacturer’s instructions [9]. The extracted DNA was then quantified using a Qubit dsDNA BR assay (Life Technologies, USA) [10] according to the manufacturer’s instructions.

### Targeted sequencing and data processing

As described previously [11], we used the two targeted sequencing assays: (1) the 295 OncoScreen panel containing whole exons of 287 genes and selected introns of 22 genes (Burning Rock Biotech Ltd., Guangzhou, China) and (2) the 1021-gene panel containing whole exons and selected introns of 288 genes and selected regions of 733 genes (Geneplus-Beijing, Beijing, China). Detailed methods for preparing the DNA, sequencing libraries, and data processing have been previously described [11].

### Statistical analysis

TMB comparison among the different subsets of patients was performed using the Wilcoxon rank-sum test. The Kaplan–Meier method was used to estimate overall survival (OS) and PFS, and differences were compared using the log-rank test. Cox proportional hazards regression analyses were used to evaluate the independently predictive factors of each biological and clinical features associated with OS and/or PFS. Statistical significance was defined as a two-tailed *P*-value of < 0.05. All the statistical analyses were performed using IBM SPSS V.25.0 (Chicago, Armonk, NY, USA).

## Results

### Patients’ characteristics

In total, 112 patients with MM who underwent tumor sequencing were identified: 86 patients (76.8%) with primary tumors, 17 (15.2%) with metastatic tumors, and 9 (8.0%) with recurrent tumors. A total of 51 patients underwent 295-gene panel sequencing, and 61 underwent 1021-gene panel sequencing for further analysis (Supplementary Table S1). Table 1 summarizes the clinicopathological characteristics of the 112 patients with MM with a median age of 56 years (range 23–82 years); 56.3% were women and 43.8% were men. The anatomic sites of the MM were the head and neck in 33.9% of the patients, genitourinary tract in 19.6%, anorectum in 18.8%, esophagus in 17.0%, uvea in 8.9%, and small bowel in 1.8%. Histologically, 79 cases (70.5%) showed an epithelioid morphology, 8 (7.1%) showed a spindle morphology, and 21 (18.8%) showed a mixed morphology. Forty-six of the 112 (41.1%) patients with clinical stage were unknown due to the unavailability of invasion depth. The median PFS and follow-up times were 7.5 and 23.9 months, respectively. For treatment, 106 patients underwent surgical operations with a gross resection of primary or recurrent lesions (106/112, 94.6%), and 92 patients received various adjuvant treatments, including chemotherapy, radiotherapy, targeted therapy, and/or ICIs (92/112, 82.1%). Importantly, 67.0% (75/112) patients received ICIs and 32.1% (36/112) did not (Table 1).

**Table 1:** Clinicopathologic characteristics of 112 MM patients

Variable	No. of patients	%
**Total**	112	
**Gender**		
Female	63	56.3
Male	49	43.8
**Age, years**		
Median	56	
Range	23–82	
**Mitotic activity (n/mm** ^**2**^ **)**		
Median	3	
Range	0–27	
Unknown	11	
**Anatomic site**		
Head and neck ^a^	38	33.9
Genitourinary tract	22	19.6
Anorectum ^b^	21	18.8
Esophagus	19	17.0
Uvea	10	8.9
Small bowel	2	1.8
**Morphology**		
Epithelioid-cell	79	70.5
Spindle-cell	8	7.1
Mixture	21	18.8
Unknown	4	3.6
**Sample origins**		
Primary	86	76.8
Recurrent	9	8.0
Metastatic	17	15.2
**Stage** ^d^		
I	13	11.6
II	20	17.9
III	18	16.1
IV	15	13.4
Unknown	46	41.1
**ICIs received**		
With	75	67.0
Without	36	32.1
Unknown	1	0.9

**Abbreviations** MM, mucosal melanomas; ICI, immuno-checkpoint inhibitor; ^a^ Includes Dacryocyst (3, 2.7%), Conjunctiva (1, 0.9%), Nasal Cavity (16, 14.3%), Paranasal Sinus (2, 1.8%), Nasopharynx (2, 1.8%), Gumline (9, 0.8%), Cavioris Bucca (1, 0.9%), Mandible (3, 2.7%), Salivary Glands (1, 0.9%); ^b^ Rectum (15, 13.4%), Anal Cana (6, 5.4%); ^c^ Cervix (2, 1.8%), Vagina (19, 17.0%), Clitoris (1, 0.9%); ^d^ Proposed by Jun Guo in 2022

### TMB in MM

**Fig. 1:**
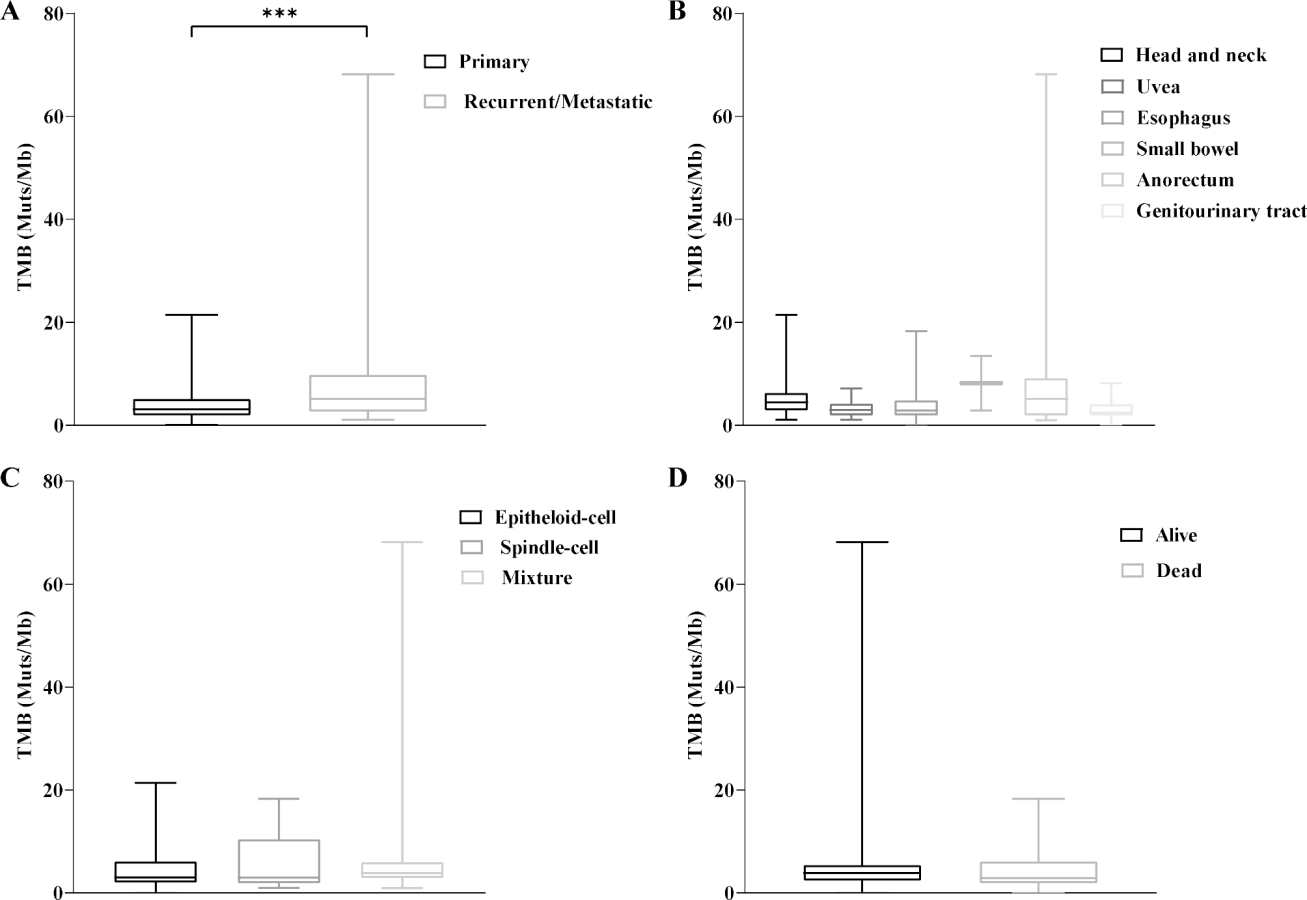
Box plots showing the differences in TMB among different clinicopathological characteristics. Box plots (**A**-**D**) are shown for TMB in sample origins (*P* = 0.015), tumor site (**B**), morphology (**C**) and patients status (**D**). Significance shown as *** P < 0.001. **Abbreviations**: TMB, tumor mutational burden

Detailed information on the genetic variation in MM is provided in Supplementary Table S2. The TMB per megabase was relatively low, with a median of 3.1 (range 0–68.2). A significantly lower TMB was observed in primary tumor samples than in recurrent/metastatic tumors (Wilcoxon rank-sum test, *P* = 0.015, Fig. 1A). There were no significant differences among the primary sites, morphological types, and survival status (Fig. 1B-D).

### Genetic profiling and related pathways of MM

**Fig. 2:**
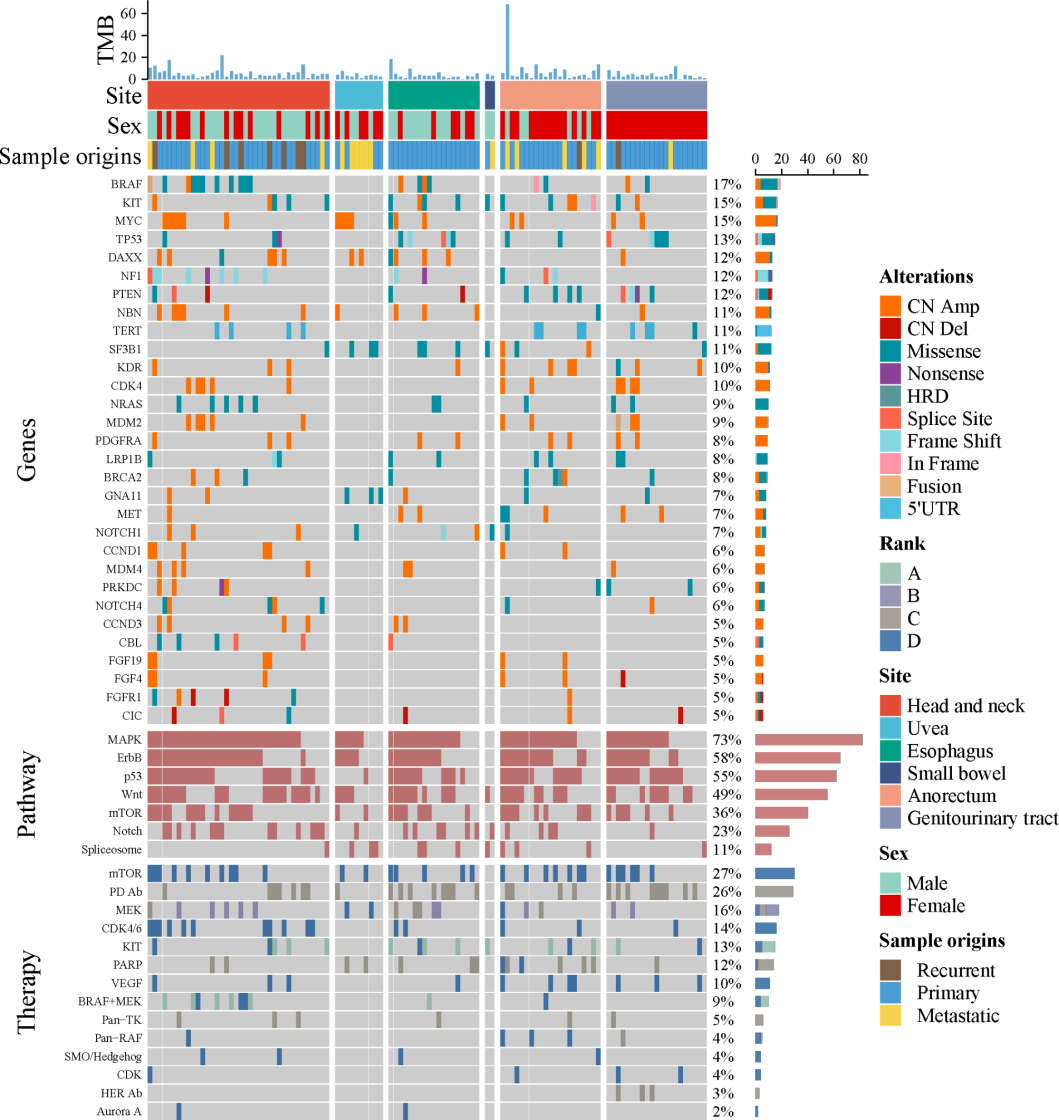
The mutational landscape of 112 patients with MM, including point mutation and SVs. From top to bottom: the total tumor mutational burden; the clinicopathological features, such as anatomic site, sex, and sample origins; the landscape of genes mutation (copy number variations, SNVs, indels, fusion gene, 5’UTR); the significantly activated pathway; the potential targeted therapy, each actionable mutation is colored by evidence level: **A** (NCCN guidelines and FDA guidelines), **B** (late trials), **C** (early trials), **D** (case report). **Abbreviations**: MM, mucosal melanoma; SVs, structural variants; SNVs, single nucleotide variations; UTR, untranslated regions; NCCN, national comprehensive cancer network; FDA, Food and Drug Administration

The frequent mutations were sequentially observed in *BRAF* (19/112, 17%), *KIT* (17/112, 15%), *MYC* (17/112, 15%), *RAS* (16/112, 14%), *TP53* (15/112, 13%), *NF1* (13/112, 12%), *SF3B1* (12/112 11%), *TERT* (11/112, 10%), *GNA11* (8/112, 7%), *CBL* (7/112, 6%), *GNAQ* (6/112, 5%) and *FBXW7* (5/112, 4%) (Fig. 2). Furthermore, according to the Kyoto Encyclopedia of Genes and Genomes (KEGG) database, the most significantly altered pathway was the MAPK signaling pathway (73%), followed by the ErbB (58%), p53 (55%), Wnt (49%), mTOR (36%), and Notch (23%) signaling pathways (Fig. 2).

**Fig. 3:**
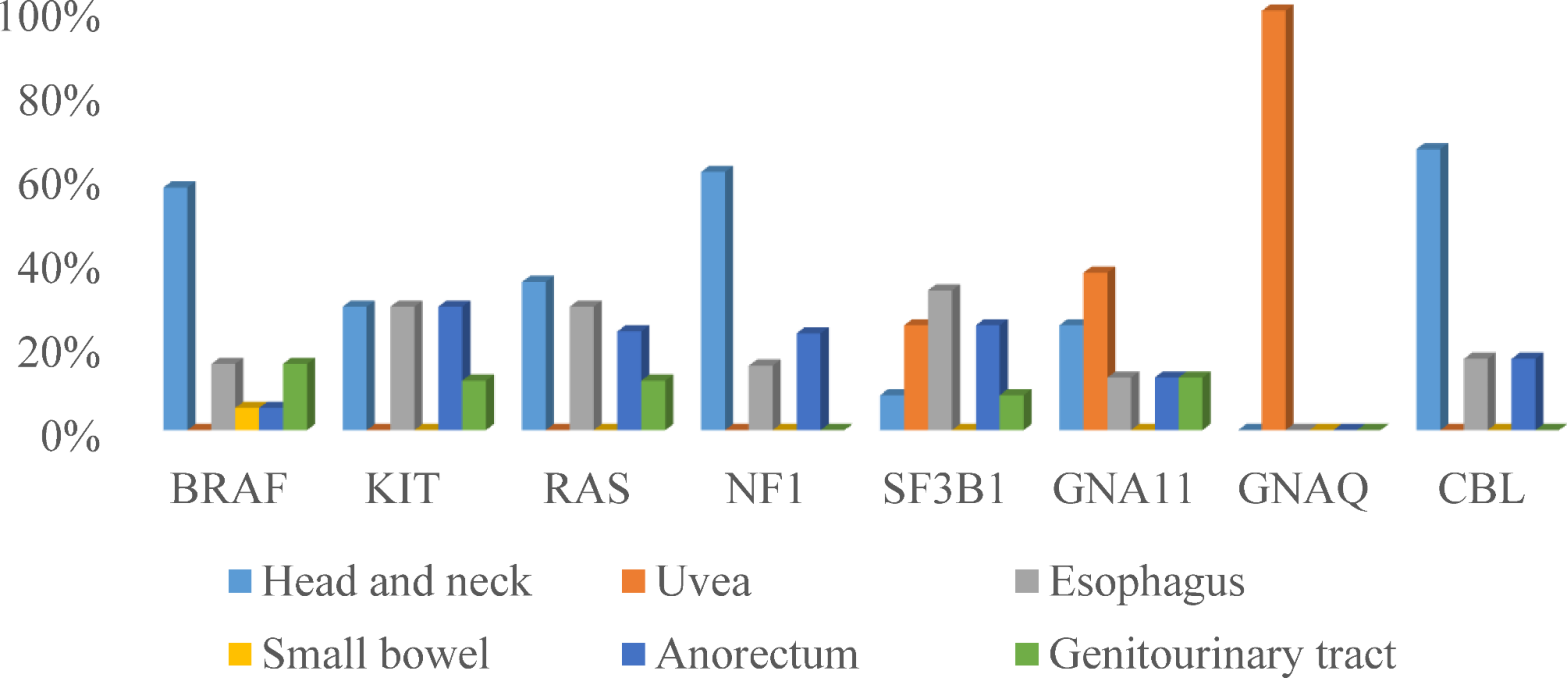
Recurring mutated genes (*BRAF*, *KIT*, *RAS*, *SF3B1*, *GNA11*, and *GNAQ*) of MM in different anatomic sites. **Abbreviations**: MM, mucosal melanoma

The frequencies of these common mutations varied according to the origin of MM. Higher percentages of *BRAF*, *RAS*, *NF1*, and *CBL* mutations were observed in patients with melanoma in the head and neck than in patients with melanoma in the other five sites. Additionally, higher percentages of *GNAQ* and *GNA11* mutations were observed in patients with uveal melanoma, and a higher percentage of *SF3B1* mutations in patients with esophageal and uveal melanoma (Fig. 3, Supplementary Figure S1).

Additionally, previous studies reported that other genes are frequently mutated in melanoma and other cancer types [4, 12–14], including *BRCA2* (9/112, 8%), *LRP1B* (9/112, 8%), *MET* (8/112, 7%), *PRKDC* (7/112, 6%), *NOTCH4* (7/112, 6%), *CCND3* (6/112, 5%), and *FGF3/4/19* (6/112, 5%). Germline mutations were observed in two cases (2/112, 2%) with *BRCA2* (p.W1692Mfs*3) and *ATM* (c.331 + 5G > A).

### Somatic copy number and structural events

Next, we observed that MM had a large number of chromosomal SVs, including somatic CN changes, fusions, and the telomere length-associated genes *TERT* and *ATRX*.

Somatic CN amplification was observed in 143 genes from 85 different chromosomal regions in 73 patients (Supplementary Figure S2). The frequently amplified regions included 6p21 (18/73, 25%), 8q24 (17/73, 23%), 4q12(14, 19%), 8q21 (11/73, 15%), 12q14 (11/73, 15%), 12q15 (10/73, 14%), 11q13 (9/73, 12%), 1q32 (8/73, 11%), and 7q31 (6/73, 8%), along with the recurrently amplified genes *MYC* (16/73, 22%), *KIT* (13/73, 28%), *DAXX* (11/73, 15%), *KDR* (11/72, 15%), *NBN* (11/72, 15%), *CDK4* (11/72, 15%), *MDM2* (10/73, 14%), *PDGFRA* (9/73, 12%), *CCND1* (7/73, 10%), *MDM4* (7/73, 10%), *CCND3* (6/73, 8%), *FGF19* (6/73, 8%), *MET* (6/73, 8%), *FGF3* (5/73, 7%), *FGF4* (5/73, 7%), *FANCE* (5/73, 7%), *IRF4* (5/73, 7%), *CDK6* (5/73, 7%), *BRAF* (5/73, 7%), *NOTCH1* (4/73, 5%), and *NOTCH2* (4/73, 5%). We also found several CN variations and the co-occurrence of *KIT*, *KDR*, *PDGFRA*, *FGF3*/*4*/*19*, *FANCE*, *DAXX*, and *NOTCH* clustered in the same chromosomal segment in one case, whereas CN deletions were detected in 14 cases. For example, *CIC* and *PTEN* deletions were more frequent in three and two cases, respectively.

Novel, recurrently fused genes were observed in nine cases (9/112, 8%), among which *RNF43*-*IFLTD1*, *KCTD15*-*BRAF*, and *WDR64*-*AKT3* had a higher mutational abundance of 25%. The last two fusion genes encode tyrosine kinases, which may activate the MAPK pathway [15].

Finally, highly prevalent *TERT* mutations were identified in 11 cases (11/112, 10%), all with *TERT* promoter mutations containing c.146 C > T (4/11, 36%), c.124 C > T (4/11, 36%), c.-124 C > T (2/11, 18%), and c.-58-u68_-58-u66delCCCinsTCT (1/11,9%). Most *ATRX* variations had single nucleotide alterations or small fragment deletions. Loss of function was only detected in six cases (6/112, 5%). Interestingly, none of the cases had mutations in either *TERT* or *ATRX*, indicating they are mutually exclusive in MM, similar to those observed in gliomas [16].

### Patients’ survival and clinical response to ICIs

**Fig. 4:**
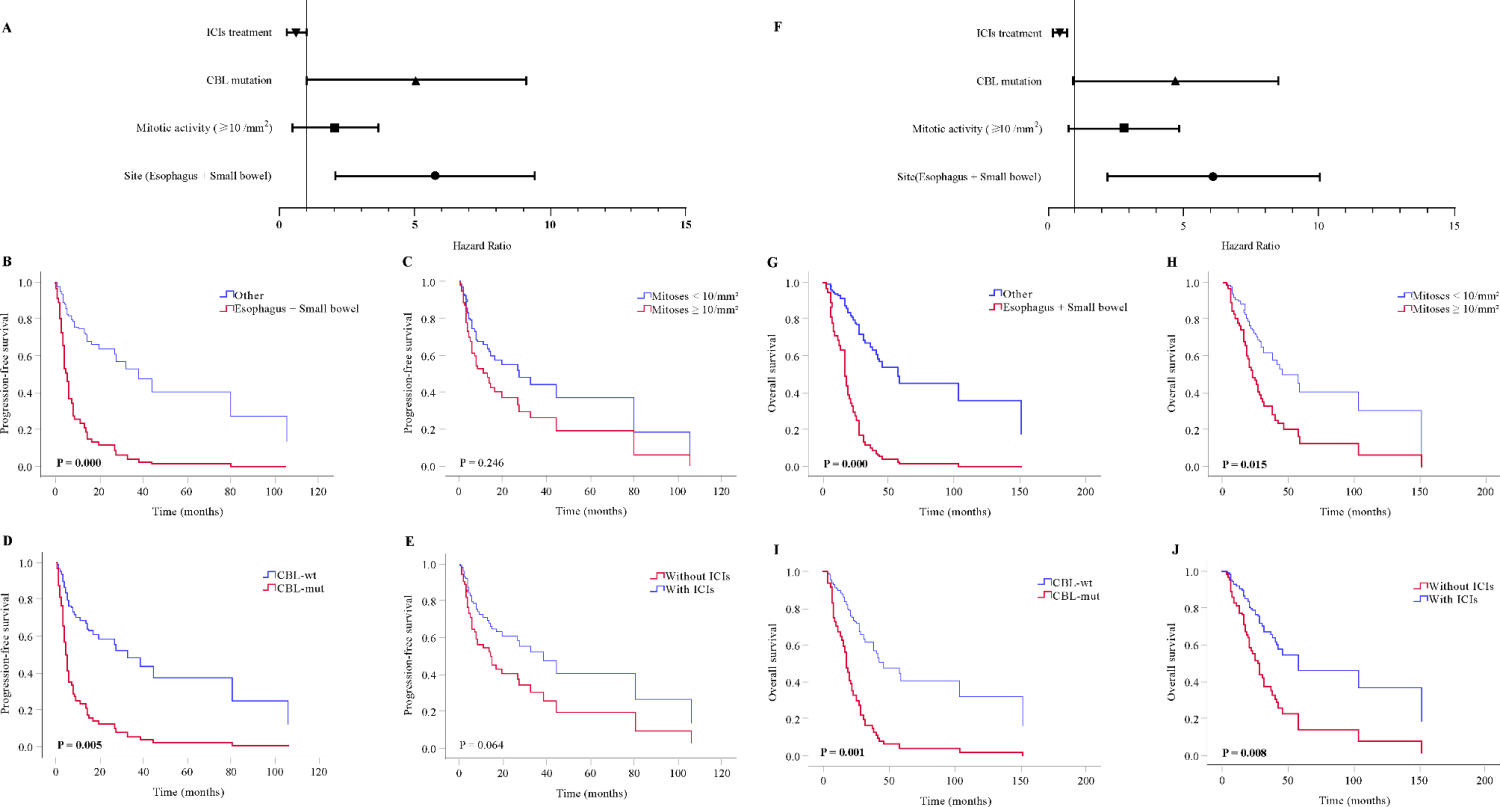
Kaplan–Meier survival analysis in MM. Forest plot and Kaplan–Meier survival curves for PFS (**A**-**E**) and OS (**F**-**J**) were performed for the primary tumor site, mitotic activity, *CBL* mutations, and with/without ICIs treatment. Significance shown as P < 0.05. **Abbreviations**: MM, mucosal melanoma; PFS, progression-free survival; OS, overall survival; *CBL*, Casitas B-lineage lymphoma; ICI, immune checkpoint inhibitor

As shown in Table 2; Fig. 4, multivariate and Kaplan–Meier analyses revealed that the anatomic sites of MM in the esophagus and small bowel (PFS: hazard ratio [HR] = 4.78, 95% confidence interval [CI] = 2.42–9.45, *P* < 0.001; OS: HR = 5.26, 95% CI = 2.51–11.03, *P* < 0.001) and *CBL* mutations (PFS: HR = 3.54, 95% CI = 1.46–8.56, *P* = 0.005; OS: HR = 5.57, 95% CI = 2.04–15.19, *P* = 0.001) were independently risk factors for survival. In addition, we found that mitotic activity with ≥ 10/mm^2^ (HR = 2.76, 95% CI = 1.22–6.25, *P* = 0.015) was an independent risk factor for OS. There were no associations between other factors and PFS or OS (Supplementary Figures S3 and S4).

**Table 2:** Univariate and multivariate Cox Regression analyses for PFS and OS in MM

Variable	PFS						OS					
	Univariate			Multivariate			Univariate			Multivariate		
	HR	95.0% CI	P^a^	HR	95.0% CI	P^a^	HR	95.0% CI	P^a^	HR	95.0% CI	P^a^
Gender (Female)	1.21	0.66–2.23	0.542				1.30	0.71–2.36	0.394			
Age (≥ 56 years)	0.76	0.41–1.39	0.371				0.89	0.49–1.61	0.702			
Site (Esophagus)	5.24	2.30-11.92	0.000				6.50	2.84–14.87	0.000			
Site (Small bowel)	9.74	1.20–79.60	0.034				3.92	0.50-31.27	0.180			
Site (Esophagus + Small bowel)	4.98	2.53–9.78	0.000	4.78	2.42–9.45	0.000	5.27	2.68–10.36	0.000	5.26	2.51–11.03	0.000
Stage (III - IV)	1.46	0.70–3.06	0.314				1.23	0.61–2.51	0.570			
Mitotic activity (≥ 3 /mm^2^)	1.12	0.57–2.19	0.744				1.22	0.64–2.34	0.547			
Mitotic activity (≥ 10 /mm^2^)	1.65	0.71–3.84	0.246				2.30	1.05–5.06	0.039	2.76	1.22–6.25	0.015
Morphology (Spindle cell)	0.61	0.18–2.07	0.432				0.58	0.18–1.93	0.377			
Ulceration	1.22	0.15–9.80	0.849				0.82	0.22–3.13	0.773			
BRAF mutation	1.26	0.56–2.87	0.578				1.14	0.51–2.56	0.754			
KIT mutation	0.91	0.43–1.90	0.793				1.18	0.56–2.48	0.661			
RAS mutation	1.50	0.66–3.38	0.339				1.32	0.59–2.97	0.450			
TP53 mutation	1.14	0.45–2.92	0.784				0.96	0.41–2.27	0.923			
NF1 mutation	1.42	0.65–3.11	0.380				1.38	0.63–3.02	0.420			
SF3B1 mutation	0.70	0.24–1.99	0.498				0.93	0.32–2.68	0.897			
FBXW7 mutation	1.58	0.55–4.51	0.397				1.53	0.55–4.31	0.418			
CBL mutation	3.96	1.65–9.52	0.002	3.54	1.46–8.56	0.005	3.70	1.54–8.90	0.004	5.57	2.04–15.19	0.001
TERT mutation	1.24	0.44–3.50	0.687				1.80	0.39–3.05	0.880			
ATRX mutation	1.64	0.58–4.63	0.348				1.46	0.52–4.09	0.475			
ICIs treatment ^b^	0.56	0.30–1.04	0.064				0.40	0.22–0.75	0.004	0.39	0.20–0.75	0.008

**Abbreviations** CI, confidence interval; HR, hazard ratio; PFS, progression-free Survival; OS, overall survival; MM, mucosal melanomas; CBL, Casitas B-lineage lymphoma; ICI, immune checkpoint inhibitor; ^a^*P* values were from Cox proportional hazard regression models; ^b^ indicates that patients received ICIs throughout the treatment for OS while before the progression for PFS

The suggested therapy modalities are listed in the bottom panel of Fig. 2, and mTOR, PD1, *MEK*, *CDK4*/*6*, *KIT, PARP, VEGF, BRAF* inhibitors may have pharmacological effects on targetable treatment [17–21]. In our study, we found that receiving ICIs (HR = 0.39, 95% CI = 0.20–0.75, *P* = 0.005, Table 2; Fig. 4J) was a favorable factor for OS (Table 2), suggesting that patients with MM may benefit from ICIs treatment.

## Discussion

In this retrospective cohort study, we used two commercially customized NGS panels to describe the molecular spectrum of 112 MM patients and explore potential targets for prognosis prediction and further individualized immunotherapy in these patients.

Consistent with previous reports, which revealed that acral melanoma and MM had limited DNA mutational burden [14, 22–24], we observed a low TMB in this study. As previously reported [4, 14, 25–27], we found that a substantial proportion of MM had alterations in the genes involved in the MAPK pathway, including frequent mutations in *BRAF*, *KIT, RAS*, and *NF1.* It is well known that a *BRAF* mutation provides a targeted therapy for melanoma with good efficacy [28]. Nevertheless, small bowel melanoma in this study harbored only two in-frame mutations in *BRAF* (p.T599_V600insT and p.T244_L245delinsS), whereas no *V600* mutations were observed. Mutations in other genes, such as *KIT*, *RAS*, and *NF1*, were found in the primary sites of the head and neck, esophagus, anorectum, and genitourinary tract in our cohort. Notably, we found that *SF3B1* mutations as recurrent genetic events in MM were more common in esophageal and uveal melanomas, which is similar to that reported in recent studies [29–31]. In addition, mutations in *GNAQ* and *GNA11* occurred in the majority of uveal melanomas found in this study, which is consistent with the findings of previous research [29, 32].

A previous whole-genome study showed that the SV load had the feature of complexity and diversity in MM [4]. Not surprisingly, we also found that most patients with MM harbored frequent CN variations and *MYC* amplification, which exemplified their role in MM pathogenesis and was in line with the results of other investigations [4, 33, 34]. For instance, high *MYC* expression is associated with aggressive biological behavior in cancers [35]. Additionally, many of the CN amplifications in well-known pathogenic genes (*KIT*, *PDGFRA*, *MDM2*, and *MDM4*) and cell cycle genes, such as *CDK4/6* and *CCND1*, were found in this study, similar to those reported in previous studies [33, 34]. Bucheit et al. have demonstrated that complete *PTEN* loss correlates with poor survival in stage III melanoma [36]. In this study, we found PTEN deletion in MM, suggesting a tumorigenic role for progression in MM. The activation of *TERT* and inactivation of *ATRX* commonly and exclusively occurred in MM, which has been reported previously, indicating the importance of telomerase activation or alternative telomere lengthening mechanisms in reducing length in MM [4, 14].

A cohort of 466 Chinese patients with melanoma showed that MM was more aggressive and was associated with shorter survival than cutaneous lesions [37]. MM arises from different anatomic sites, and a population-based epidemiological analysis showed that MM from different anatomical sites exhibit different survival outcomes, likely due to the diverse environmental exposures associated with each site [38]. Here, we demonstrated that the anatomic sites of esophagus and small bowel melanomas may be a risk factor for survival, which concurs with the findings of a previous study [39]. However, a cohort of 706 MM patients did not show significant differences in prognosis among the anatomic sites of MM, primarily due to lack of cases of MM in the esophagus and small bowel [40].

We identified *CBL* mutations in patients with MM; these were risk factors for poor OS and PFS, which are infrequent in published melanoma exome studies but frequent in desmoplastic melanoma [41]. Recurrent *CBL* mutations occur in myeloid malignancies and have been associated with poor prognosis [42]. Ebert et al. recently identified that the oncogenic function of *CBL* mutants drive PI3K/AKT signaling and provide a rationale for therapeutic targets in myeloid malignancies [43]. Another study identified that inhibiting *CBL* mutations can activate the innate immune system to restrain cancer metastasis and improve the sensitivity to immunotherapy [44]. Finally, we found that MM patients receiving ICI treatment experienced a favorable impact on survival, especially OS, which is consistent with the findings of a previous study [45]. Taken together, *CBL* mutations might be promising targets for MM immunotherapy.

Our study had some limitations. First, the sample size was small owing to the rarity of this disease, especially small bowel melanoma. Second, for molecular profiling in MM, we used targeted NGS, which only targeted genomic regions of partial genes and, therefore, could not reveal novel pathogenic point mutations, rearrangements, or epigenetic changes. Thus, the whole-exome sequencing in a larger cohort is required to comprehensively depict the molecular profile of MM. A better understanding of the molecular mechanisms is required to explore additional avenues for immunotherapy in MM.

## Conclusions

Our study demonstrates the molecular landscape of Chinese patients with MM based on targeted sequencing. Our finding on the genetic characteristics of MM among different anatomic sites reveal that *CBL* mutations in MM are potential targets. Further studies are warranted to elucidate the mechanisms that link *CBL* mutations to immunotherapy responses, which may provide a rationale for immunotherapy.

### Electronic supplementary material

Below is the link to the electronic supplementary material.

Supplementary Material 1

Supplementary Material 2

Supplementary Material 3

Supplementary Material 4

Supplementary Material 5

Supplementary Material 6

## Data Availability

The key raw data were deposited into the Research Data Deposit, with the approval number of RDDA2022535268, and the datasets used in this study are publicly available.
