# Supplementary material for: Clinical significance of genetic profiling based on different anatomic sites in patients with mucosal melanoma who received or did not receive immune checkpoint inhibitors
Source: Cancer Cell Int. 2023 Aug 30;23:187. doi: 10.1186/s12935-023-03032-3 (PMC10469937; doi:10.1186/s12935-023-03032-3)
Supplement: Supplementary file 1 — Supplementary Material 1 [file 12935_2023_3032_MOESM1_ESM.pdf]

Supplementary Table S1. Detailed clinicopathological features of the 112 MM patients

Case No.	NGS Panel	Gender	Age	Smoking (years)	Site	Detailed location	Sample Origins	Stage	Morphology	Mitotic activity (n/mm^{2})	Ulceration	Pigmentation	Lymphovascular invasion	Plasma LDH	Specimen type	ICIs	Recurrence/metastasis	PFS (months)	OS (months)	Status
1	295	Male	54	30	Head and neck	Nasal cavity	Recurrent	Unknown	Epithelioid cell	3	Unknown	Yes	Unknown	Elevated	Operation	Pembrolizumab	M (lung)	26.37	39.20	Alive
2	295	Female	60	0	Head and neck	Nasal cavity	Primary	IV	Epithelioid cell	Unknown	Unknown	Yes	Unknown	Elevated	Operation	Without	M (lung)	14.53	31.80	Dead
3	295	Male	64	40	Head and neck	Nasal cavity	Recurrent	Unknown	Spindle cell	7	Yes	Yes	Yes	Unknown	Operation	Nivolumab;Ipilimumab	R	105.60	151.27	Dead
4	295	Male	53	0	Head and neck	Nasal cavity	Primary	IV	Epithelioid cell	1	Unknown	Yes	Unknown	Normal	Operation	Toripalimab	M (bone)	19.37	21.33	Alive
5	295	Female	69	0	Head and neck	Nasal cavity	Primary	III	Epithelioid cell	7	Unknown	Unknown	Unknown	Normal	Operation	Without	R	43.40	44.43	Alive
6	295	Male	63	30	Head and neck	Nasal cavity	Primary	IV	Epithelioid cell	4	Unknown	Yes	Unknown	Normal	Operation	Without	M (lung)	6.33	27.23	Alive
7	295	Male	55	0	Head and neck	Nasal cavity	Primary	IV	Epithelioid cell	7	Unknown	No	Unknown	Normal	Operation	Pembrolizumab	M (lung)	34.93	54.53	Alive
8	295	Female	66	0	Head and neck	Nasal cavity	Recurrent	Unknown	Epithelioid cell	3	Unknown	Yes	Unknown	Unknown	Operation	Pembrolizumab	M (breast)	7.83	7.83	Alive
9	295	Female	28	0	Head and neck	Nasal cavity	Primary	III	Epithelioid cell	3	No	Yes	Unknown	Elevated	Operation	Pembrolizumab	M (lung)	22.00	47.57	Alive
10	295	Female	53	0	Head and neck	Nasal cavity	Primary	IV	Epithelioid cell	11	Unknown	Yes	Yes	Elevated	Operation	Pembrolizumab	M (LN)	13.30	31.00	Dead
11	1021	Male	75	0	Head and neck	Mandible	Primary	IV	Unknown	Unknown	Unknown	Yes	Yes	Normal	Operation	Pembrolizumab	M (lung)	0.40	10.07	Alive
12	1021	Female	66	0	Head and neck	Nasal cavity	Primary	III	Epithelioid cell	Unknown	Unknown	Yes	Unknown	Normal	Operation	Without	M (LN)	9.40	20.00	Alive
13	1021	Male	74	0	Head and neck	Nasal cavity	Recurrent	IV	Mixed[a]	1	Unknown	Yes	Unknown	Normal	Operation	Pembrolizumab	R	24.63	34.43	Alive
14	1021	Female	58	0	Head and neck	Nasal cavity	Primary	IV	Epithelioid cell	15	Unknown	Yes	Unknown	Normal	Operation	Without	Unknown	7.50	7.50	Alive
15	1021	Male	42	20	Head and neck	Nasal cavity	Primary	IV	Mixed	1	No	Yes	Yes	Normal	Operation	Toripalimab	M (liver)	1.43	36.23	Alive
16	295	Female	52	0	Head and neck	Nasal cavity	Primary	Unknown	Mixed	2	Unknown	No	Unknown	Normal	Operation	Toripalimab	R	1.10	5.97	Dead
17	295	Male	29	0	Head and neck	Nasopharynx	Primary	IV	Epithelioid cell	9	Unknown	Yes	Unknown	Normal	Operation	Pembrolizumab	M (liver)	11.17	53.70	Alive
18	295	Male	82	0	Head and neck	Nasopharynx	Recurrent	Unknown	Epithelioid cell	1	Unknown	Yes	Unknown	Normal	Operation	Pembrolizumab	R	41.83	60.43	Alive
19	295	Male	43	0	Head and neck	Conjunctiva	Metastatic (LN)	Unknown	Epithelioid cell	3	Unknown	No	Unknown	Normal	Operation	Nivolumab;Pembrolizumab	M (LN)	24.33	69.73	Alive
20	295	Female	54	0	Head and neck	Dacryocyst	Recurrent	Unknown	Epithelioid cell	3	Unknown	Yes	Unknown	Normal	Biopsy	Pembrolizumab;Nivolumab;Ipilimumab	R	4.00	27.57	Dead
21	295	Male	56	0	Head and neck	Dacryocyst	Recurrent	IV	Mixed	13	Unknown	Yes	Unknown	Normal	Operation	Pembrolizumab	R	11.23	37.27	Alive
22	295	Male	44	0	Uvea	Choroid	Metastatic (tonsil)	Unknown	Epithelioid cell	9	Unknown	Yes	Unknown	Normal	Operation	Without	M (intestine)	26.67	45.87	Dead
23	295	Female	57	0	Uvea	Choroid	Metastatic (liver)	Unknown	Spindle cell	2	Unknown	Yes	Unknown	Elevated	Biopsy	Pembrolizumab	M (liver)	51.70	59.13	Alive
25	295	Female	55	0	Uvea	Choroid	Metastatic (liver)	IV	Mixed	2	Unknown	Yes	Unknown	Normal	Biopsy	Pembrolizumab	M (liver)	44.07	57.30	Dead
26	295	Male	45	0	Uvea	Choroid	Metastatic (liver)	IV	Epithelioid cell	1	Unknown	Yes	Unknown	Normal	Biopsy	Pembrolizumab	M (liver)	80.30	103.53	Dead
27	1021	Female	50	0	Uvea	Choroid	Primary	Unknown	Spindle cell	1	Unknown	Yes	Unknown	Elevated	Operation	Toripalimab;Camrelizumab	M (liver)	6.17	31.27	Alive
28	295	Female	78	0	Head and neck	Cavioris bucca	Primary	IV	Epithelioid cell	10	Unknown	No	Yes	Normal	Operation	Nivolumab;Pembrolizumab	M (liver)	2.13	43.63	Alive
29	1021	Male	48	0	Head and neck	Gumline	Primary	Unknown	Spindle cell	3	Unknown	Yes	No	Normal	Operation	Pembrolizumab	Unknown	5.10	5.10	Alive
30	1021	Male	54	15	Head and neck	Mandible	Metastatic (LN)	Unknown	Mixed	9	Yes	Yes	Yes	Normal	Operation	Pembrolizumab	R	11.87	25.77	Alive
31	295	Male	38	0	Head and neck	Mandible	Primary	Unknown	Epithelioid cell	2	Unknown	Yes	Unknown	Normal	Operation	Pembrolizumab	R	7.13	50.90	Alive
32	1021	Male	71	0	Head and neck	Gumline	Primary	Unknown	Mixed	16	Yes	Yes	No	Normal	Operation	Without	R	7.93	9.00	Alive
34	295	Male	53	0	Head and neck	Gumline	Primary	Unknown	Epithelioid cell	2	Unknown	Yes	Unknown	Normal	Operation	Without	M (LN)	5.47	17.60	Dead
35	295	Male	66	0	Head and neck	Gumline	Metastatic (lung)	Unknown	Epithelioid cell	2	Unknown	Yes	Yes	Normal	Biopsy	Pembrolizumab	M (lung)	27.40	58.30	Dead
36	295	Male	53	0	Head and neck	Gumline	Primary	IV	Spindle cell	2	Unknown	No	Yes	Normal	Operation	Pembrolizumab	M (LN)	0.53	28.03	Dead
37	295	Female	56	0	Head and neck	Gumline	Primary	Unknown	Epithelioid cell	7	Yes	Yes	Yes	Normal	Operation	Without	Unknown	22.77	22.77	Alive
38	1021	Male	57	0	Head and neck	Gumline	Metastatic (pancreas)	Unknown	Mixed	0	Yes	Yes	Yes	Normal	Operation	Pembrolizumab;Toripalimab;Ipilimumab	M (pancreas)	13.23	53.57	Alive
39	295	Male	50	30	Head and neck	Gumline	Primary	Unknown	Epithelioid cell	3	Unknown	Yes	Yes	Normal	Biopsy	Pembrolizumab	M (brain)	16.63	23.17	Dead
40	1021	Male	34	0	Head and neck	Salivary glands	Primary	Unknown	Epithelioid cell	1	Unknown	Yes	Yes	Normal	Operation	Toripalimab	M (lung)	3.37	16.07	Dead
41	295	Male	54	0	Head and neck	Paranasal sinus	Primary	Unknown	Epithelioid cell	1	Unknown	Yes	Unknown	Normal	Operation	Without	R	19.03	41.80	Alive
42	295	Female	67	0	Head and neck	Paranasal sinus	Primary	III	Epithelioid cell	10	Unknown	Yes	Unknown	Normal	Operation	Pembrolizumab	M (liver)	1.50	6.00	Dead
43	1021	Female	69	0	Esophagus	Esophagus	Primary	III	Epithelioid cell	4	Unknown	Yes	Yes	Elevated	Operation	Pembrolizumab	M (LN)	1.60	11.37	Dead
44	1021	Male	58	30	Esophagus	Esophagus	Primary	I	Epithelioid cell	3	Unknown	Yes	No	Normal	Operation	Pembrolizumab	M (LN)	8.60	18.17	Dead
45	1021	Male	63	0	Esophagus	Esophagus	Primary	Unknown	Epithelioid cell	1	Unknown	Yes	Unknown	Unknown	Biopsy	Without	Unknown	NA	NA	Alive
46	1021	Male	56	0	Esophagus	Esophagus	Primary	III	Mixed	9	Yes	Yes	Yes	Normal	Operation	Without	R	4.83	22.57	Alive
48	1021	Female	64	0	Esophagus	Esophagus	Primary	III	Epithelioid cell	6	Unknown	Yes	Yes	Normal	Operation	Without	M (LN)	3.53	7.23	Dead
49	1021	Male	58	0	Esophagus	Esophagus	Primary	Unknown	Spindle cell	5	Unknown	Yes	Unknown	Elevated	Biopsy	Without	M (LN)	5.77	13.57	Dead
50	1021	Male	62	23	Esophagus	Esophagus	Primary	I	Epithelioid cell	3	Unknown	Yes	Unknown	Normal	Operation	Without	Unknown	NA	NA	Dead
52	295	Male	59	0	Esophagus	Esophagus	Primary	III	Epithelioid cell	2	Unknown	Yes	Yes	Normal	Operation	Pembrolizumab	M (LN)	7.07	22.27	Dead
53	1021	Female	58	0	Esophagus	Esophagus	Primary	IV	Epithelioid cell	1	Unknown	Yes	Yes	Elevated	Biopsy	Without	M (liver)	NA	9.57	Alive
54	295	Male	45	0	Esophagus	Esophagus	Primary	Unknown	Epithelioid cell	1	Unknown	Yes	Unknown	Unknown	Operation	Without	M (cutaneous)	3.07	16.57	Dead
55	1021	Male	56	0	Esophagus	Esophagus	Primary	I	Epithelioid cell	3	Unknown	No	No	Normal	Operation	Without	M (lung)	6.13	9.27	Dead
56	295	Male	47	10	Esophagus	Esophagus	Primary	III	Mixed	2	Unknown	Unknown	Yes	Normal	Operation	Toripalimab	M (lung)	19.67	42.27	Dead
57	295	Male	47	30	Esophagus	Esophagus	Primary	III	Epithelioid cell	15	Unknown	Yes	Yes	Elevated	Operation	Pembrolizumab	M (bone)	11.37	15.50	Alive
58	1021	Female	53	0	Esophagus	Esophagus	Primary	III	Mixed	1	Unknown	Unknown	Yes	Elevated	Operation	Without	M (liver)	1.13	3.40	Dead
59	295	Male	71	30	Esophagus	Esophagus	Primary	II	Epithelioid cell	1	Unknown	Yes	No	Normal	Operation	Without	M (liver)	2.07	3.77	Dead
60	295	Male	53	20	Esophagus	Esophagus	Primary	IV	Epithelioid cell	13	Unknown	Yes	Yes	Elevated	Biopsy	Pembrolizumab	General metastasis	3.53	16.60	Dead
61	1021	Male	23	0	Esophagus	Esophagus	Primary	I	Epithelioid cell	7	Unknown	Unknown	Unknown	Elevated	Operation	Without	M (LN)	4.90	17.37	Dead
62	295	Female	82	0	Esophagus	Esophagus	Primary	III	Epithelioid cell	1	Unknown	Yes	Yes	Normal	Biopsy	Nivolumab	General metastasis	2.40	6.73	Dead
63	1021	Female	58	0	Esophagus	Esophagus	Primary	I	Mixed	3	Yes	Yes	No	Normal	Operation	Pembrolizumab	M (liver)	21.73	31.43	Alive
64	1021	Male	63	0	Small bowel	Small bowel	Primary	II	Epithelioid cell	10	Yes	Yes	Unknown	Normal	Operation	Without	General metastasis	3.87	16.23	Dead
65	1021	Male	47	0	Small bowel	Small bowel	Metastatic (stomach)	II	Mixed	3	Yes	No	Unknown	Normal	Operation	Pembrolizuma	M (brain)	1.07	21.83	Alive
66	1021	Female	65	0	Anorectum	Rectum	Primary	II	Mixed	1	Yes	Yes	No	Normal	Operation	Pembrolizuma	R	16.47	36.23	Alive
67	1021	Female	65	0	Anorectum	Rectum	Primary	I	Epithelioid cell	0	Unknown	Yes	No	Normal	Operation	Without	M (lung)	32.33	37.53	Dead
68	1021	Female	56	0	Anorectum	Rectum	Primary	III	Epithelioid cell	11	Unknown	No	Yes	Elevated	Operation	Pembrolizuma； Atezolizumab	M (liver)	2.20	6.13	Dead
69	1021	Female	50	0	Anorectum	Rectum	Metastatic (liver)	III	Epithelioid cell	1	Unknown	No	Yes	Normal	Biopsy	Toripalimab	R	14.40	28.43	Dead
70	1021	Male	47	3.75	Anorectum	Rectum	Primary	Unknown	Epithelioid cell	2	Unknown	Yes	Unknown	Normal	Operation	Toripalimab	R	33.83	61.70	Alive
71	1021	Female	41	0	Anorectum	Rectum	Primary	III	Mixed	25	Yes	Unknown	Yes	Normal	Operation	Pembrolizumab	M (LN)	11.17	13.23	Dead
72	295	Female	66	0	Anorectum	Rectum	Primary	I	Epithelioid cell	0	Unknown	Yes	No	Normal	Operation	Pembrolizumab	M (cutaneous)	7.50	20.20	Dead
73	295	Male	50	0	Anorectum	Rectum	Primary	III	Epithelioid cell	6	Unknown	Yes	Yes	Normal	Operation	Without	M (liver)	5.17	7.77	Dead
74	295	Female	57	0	Anorectum	Rectum	Primary	II	Epithelioid cell	4	Yes	Yes	No	Normal	Operation	Pembrolizumab;Ipilimumab	M (lung)	13.83	20.93	Dead
75	295	Male	53	0	Anorectum	Anal cana	Primary	IV	Epithelioid cell	2	Yes	Yes	Unknown	Normal	Biopsy	Pembrolizumab	M (liver)	8.40	25.10	Dead
76	295	Female	63	0	Anorectum	Rectum	Primary	Unknown	Spindle cell	5	Unknown	Unknown	Unknown	Normal	Biopsy	Pembrolizumab		6.07	35.20	Alive
77	1021	Female	60	0	Anorectum	Rectum	Primary	II	Epithelioid cell	3	Yes	Yes	Unknown	Unknown	Operation	Without	M (LN)	23.77	24.70	Alive
78	1021	Female	62	0	Anorectum	Anal cana	Metastatic (liver)	III	Epithelioid cell	1	Unknown	Yes	Yes	Normal	Biopsy	Pembrolizumab	M (lung)	75.07	130.23	Alive
79	295	Female	41	0	Anorectum	Anal cana	Primary	III	Epithelioid cell	1	Unknown	Yes	Unknown	Normal	Operation	Nivolumab;Iplimumab	M (LN)	3.50	25.20	Dead
80	1021	Female	65	0	Anorectum	Anal cana	Metastatic (stomach)	Unknown	Epithelioid cell	Unknown	Unknown	Yes	Unknown	Unknown	Biopsy	Without	R	38.20	41.77	Dead
81	1021	Female	53	0	Anorectum	Anal cana	Primary	I	Epithelioid cell	8	Yes	Yes	No	Unknown	Operation	Nivolumab；Pembrolizumab	M (LN)	5.10	55.33	Alive
82	295	Female	56	0	Genitourinary tract	Cervix	Primary	II	Epithelioid cell	6	Unknown	Yes	No	Normal	Operation	Durvalumab;Nivolumab;Ipilimumab Pembrolizumab	M (lung)	4.43	32.50	Alive
83	1021	Female	45	0	Genitourinary tract	Vigina	Primary	II	Spindle cell	1	Unknown	Yes	No	Normal	Operation	Pembrolizumab	M (LN)	1.40	7.20	Alive
85	1021	Female	32	0	Genitourinary tract	Vigina	Primary	II	Epithelioid cell	1	No	Yes	No	Normal	Biopsy	Sintilimab； Pembrolizumab	M (liver)	13.70	19.83	Alive

86	1021	Female	51	0	Genitourinary tract	Vigina	Primary	I	Epithelioid cell	1	Unknown	Yes	Yes	Normal	Operation	Toripalimab	R	1.43	1.53	Alive
87	1021	Female	58	0	Genitourinary tract	Vigina	Primary	II	Epithelioid cell	15	Yes	Yes	No	Normal	Operation	Without	R	7.27	7.27	Alive
88	1021	Female	29	0	Genitourinary tract	Vigina	Primary	I	Mixed	25	Unknown	Yes	Unknown	Normal	Operation	Pembrolizumab；Nivolumab;Ipilimumab	M (lung and liver)	12.70	25.00	Alive
89	1021	Female	53	0	Genitourinary tract	Vigina	Primary	Unknown	Epithelioid cell	6	Yes	Yes	Unknown	Normal	Operation	Without	M (LN)	1.30	27.40	Alive
90	295	Female	60	0	Genitourinary tract	Vigina	Metastatic (brain)	I	Epithelioid cell	3	Yes	Yes	No	Normal	Operation	Nivolumab	M (liver)	57.77	116.17	Alive
91	295	Female	41	0	Genitourinary tract	Vigina	Recurrent	II	Epithelioid cell	0	Unknown	No	No	Normal	Operation	Without	Unknown	8.20	8.20	Dead
92	295	Female	70	0	Genitourinary tract	Vigina	Primary	II	Mixed	10	Yes	Yes	No	Normal	Operation	Without	R	4.67	19.40	Dead
93	295	Female	68	0	Genitourinary tract	Vigina	Primary	II	Epithelioid cell	1	Yes	Yes	No	Normal	Operation	Nivolumab;Pembrolizumab;Ipilimumab	R	3.17	50.67	Alive
94	295	Female	34	0	Genitourinary tract	Vigina	Primary	III	Epithelioid cell	9	No	Unknown	Yes	Normal	Operation	Nivolumab	M (lung)	3.30	37.40	Dead
95	295	Female	44	0	Genitourinary tract	Vigina	Primary	Unknown	Epithelioid cell	2	No	Yes	Unknown	Unknown	Biopsy	Without	Unknown	NA	26.97	Dead
96	295	Female	67	0	Genitourinary tract	Vigina	Primary	II	Epithelioid cell	27	No	Yes	Unknown	Normal	Biopsy	Without	Unknown	NA	30.73	Dead
97	295	Female	50	0	Genitourinary tract	Vigina	Primary	II	Epithelioid cell	1	Yes	No	No	Normal	Operation	Without	M (LN)	4.13	40.70	Dead
98	1021	Female	54	0	Genitourinary tract	Vigina	Primary	Unknown	Mixed	2	Unknown	Unknown	Unknown	Normal	Operation	Toripalimab	M (brain)	4.07	30.77	Alive
99	1021	Female	66	0	Genitourinary tract	Vigina	Primary	II	Mixed	9	Unknown	Yes	No	Normal	Operation	Nivolumab;Toripalimab	R	12.00	27.97	Alive
100	1021	Female	69	0	Genitourinary tract	Clitoris	Primary	III	Epithelioid cell	3	Yes	Yes	Yes	Normal	Operation	Pembrolizumab；Nivolumab；Toripalimab	R	5.70	19.77	Dead
47	1021	Female	62	0	Genitourinary tract	Vigina	Primary	I	Epithelioid cell	5	Yes	Yes	No	Normal	Operation	Without	R	3.27	12.37	Alive
101	1021	Female	51	0	Genitourinary tract	Vigina	Primary	III	Unknown	13	Unknown	Unknown	Yes	Normal	Operation	Sintilimab	R	4.00	4.00	Alive
102	1021	Female	39	0	Genitourinary tract	Vigina	Primary	I	Epithelioid cell	Unknown	No	Yes	No	Normal	Operation	Camrelizumab	R	6.73	6.73	Alive
103	1021	Female	64	0	Genitourinary tract	Cervix	Primary	II	Epithelioid cell	Unknown	Unknown	Yes	No	Normal	Operation	Toripalimab	R	9.20	9.23	Alive
104	1021	Female	64	0	Head and neck	Dacryocyst	Primary	Unknown	Epithelioid cell	1	Unknown	Unknown	Unknown	Normal	Operation	Pembrolizumab	R	7.17	13.03	Alive
105	1021	Male	62	0	Uvea	Choroid	Metastatic (bone)	Unknown	Epithelioid cell	Unknown	Yes	Unknown	Unknown	Elevated	Operation	Pembrolizumab	General metastasis	487.00	493.80	Alive
106	1021	Male	41	0	Uvea	Choroid	Metastatic (liver)	Unknown	Epithelioid cell	Unknown	Unknown	Yes	Unknown	Elevated	Biopsy	Pembrolizuma;Durvalumab	M (lung and bone)	4.00	6.43	Alive
107	1021	Female	70	0	Head and neck	Gumline	Primary	Unknown	Mixed	Unknown	Yes	Yes	Yes	Normal	Operation	Toripalimab	R	2.80	5.33	Dead
108	1021	Female	69	0	Anorectum	Rectum	Metastatic (LN)	Unknown	Epithelioid cell	Unknown	Unknown	Unknown	Unknown	Unknown	Operation	Without	M (LN)	6.27	22.27	Alive
109	1021	Male	43	0	Anorectum	Rectum	Recurrent	Unknown	Epithelioid cell	Unknown	Yes	Yes	Unknown	Elevated	Operation	Toripalimab	General metastasis	22.70	25.23	Alive
110	1021	Male	30	0	Anorectum	Anal cana	Primary	II	Epithelioid cell	10	Yes	Yes	Unknown	Normal	Operation	Toripalimab	Unknown	7.50	7.50	Alive
111	1021	Female	61	0	Uvea	Choroid	Primary	Unknown	Epithelioid cell	1	Unknown	Yes	Unknown	Unknown	Operation	Unknown	M (liver)	34.50	40.37	Alive
112	1021	Female	51	0	Uvea	Choroid	Primary	Unknown	Epithelioid cell	2	Unknown	Yes	Unknown	Unknown	Operation	Without	Unknown	8.77	8.77	Alive
113	1021	Female	57	0	Uvea	Choroid	Primary	Unknown	Epithelioid cell	1	Unknown	Yes	Unknown	Elevated	Operation	Toripalimab；pembrolizuma；Toripalimab	M (bone)	5.53	9.43	Alive
114	1021	Male	55	0	Anorectum	Rectum	Metastatic (LN)	II	Mixed	2	Unknown	Yes	No	Normal	Operation	Pembrolizuma	M (LN)	33.23	66.37	Alive
115	1021	Male	67	0	Head and neck	Nasal cavity	Primary	Unknown	Unknown	2	Unknown	Yes	Unknown	Normal	Biopsy	Pembrolizuma	R	4.47	4.70	Alive
116	1021	Female	39	0	Anorectum	Rectum	Primary	II	Unknown	2	Unknown	Yes	No	Elevated	Operation	Pembrolizuma	Unknown	21.53	21.53	Alive

Abbreviations: MM, mucosal melanoma; NA, not available; LN, lymphy node; M, metastatic; R, recurrent; PFS, progression-free survival; OS, overall survival; [a]Mixed, epithelioid and spindle cells;
