# Supplementary material for: Clinical significance of genetic profiling based on different anatomic sites in patients with mucosal melanoma who received or did not receive immune checkpoint inhibitors
Source: Cancer Cell Int. 2023 Aug 30;23:187. doi: 10.1186/s12935-023-03032-3 (PMC10469937; doi:10.1186/s12935-023-03032-3)
Supplement: Supplementary file 2 — Supplementary Material 2 [file 12935_2023_3032_MOESM2_ESM.pdf]

Supplementary Table S2. Targeted sequencing landscapes of the 112 patients with mucosal melanoma

Case No.	NGS Panel	Site	Tumor_Sample_Barcode	TMB	Hugo_Symbol	Variant_Classification	Protein_Change	Mutational abundance (%)	Transcript_name
1	295	Head and neck	AG1900809FFP	1.02	FLT4	Missense_Mutation	p.S430F	47.47	NM_182925.4
1	295	Head and neck	AG1900809FFP	1.02	CCND3	CN_Amp	NA	4.21	NM_001760.4
1	295	Head and neck	AG1900809FFP	1.02	DAXX	CN_Amp	NA	4.16	NM_001141970.1
2	295	Head and neck	AG1800300FFP	4.08	CBL	Splice_Site	c.1096-1G>C	20.86	NM_005188.3
2	295	Head and neck	AG1800300FFP	4.08	NF1	Frame_Shift	p.A399fs	10.87	NM_000267.3
2	295	Head and neck	AG1800300FFP	4.08	STK11	Missense_Mutation	p.H168R	28.13	NM_000455.4
2	295	Head and neck	AG1800300FFP	4.08	AR	Missense_Mutation	p.P286A	23.31	NM_000044.3
3	295	Head and neck	AG1800065FFP	12.24	PIK3CG	Missense_Mutation	p.P93S	30.20	NM_001282426.1
3	295	Head and neck	AG1800065FFP	12.24	ROS1	Missense_Mutation	p.S1185G	19.60	NM_002944.2
3	295	Head and neck	AG1800065FFP	12.24	CCND1	CN_Amp	NA	8.87	NM_053056.2
3	295	Head and neck	AG1800065FFP	12.24	FGF19	CN_Amp	NA	6.96	NM_005117.2
3	295	Head and neck	AG1800065FFP	12.24	FGF3	CN_Amp	NA	6.95	NM_005247.2
3	295	Head and neck	AG1800065FFP	12.24	FGF4	CN_Amp	NA	8.69	NM_002007.2
3	295	Head and neck	AG1800065FFP	12.24	RPTOR	CN_Amp	NA	3.90	NM_020761.2
3	295	Head and neck	AG1800065FFP	12.24	FGFR3	Splice_Site	p.K310K	24.20	NM_000142.4
3	295	Head and neck	AG1800065FFP	12.24	IKBKE	Splice_Site	NA	25.60	NM_014002.3
3	295	Head and neck	AG1800065FFP	12.24	TSC2	Missense_Mutation	p.S867G	20.20	NM_000548.4
3	295	Head and neck	AG1800065FFP	12.24	ARID1A	Missense_Mutation	p.G1295E	24.70	NM_006015.4
3	295	Head and neck	AG1800065FFP	12.24	NF1	Frame_Shift	p.S1331fs	24.10	NM_000267.3
3	295	Head and neck	AG1800065FFP	12.24	NF1	Missense_Mutation	p.K2593M	28.30	NM_000267.3
3	295	Head and neck	AG1800065FFP	12.24	FGFR1	Missense_Mutation	p.G383R	36.10	NM_023110.2
3	295	Head and neck	AG1800065FFP	12.24	SETD2	Missense_Mutation	p.Q2290L	20.30	NM_014159.6
3	295	Head and neck	AG1800065FFP	12.24	KDR	CN_Amp	NA	4.61	NM_002253.2
3	295	Head and neck	AG1800065FFP	12.24	KIT	CN_Amp	NA	4.81	NM_000222.2
3	295	Head and neck	AG1800065FFP	12.24	PDGFRA	CN_Amp	NA	6.54	NM_006206.4
3	295	Head and neck	AG1800065FFP	12.24	KDM5C	Missense_Mutation	p.G1198E	57.50	NM_004187.3
3	295	Head and neck	AG1800065FFP	12.24	PTEN	Missense_Mutation	p.S10R	35.30	NM_000314.6
3	295	Head and neck	AG1800065FFP	12.24	KAT6A	CN_Amp	NA	3.87	NM_006766.4
4	295	Head and neck	AG1800296FFP	3.06	PTEN	Splice_Site	c.210-2A>G	63.65	NM_000314.6
4	295	Head and neck	AG1800296FFP	3.06	RET	CN_Del	NA	1.04	NM_020975.4
4	295	Head and neck	AG1800296FFP	3.06	CIC	CN_Del	NA	0.45	NM_015125.4
4	295	Head and neck	AG1800296FFP	3.06	PPP2R1A	CN_Amp	NA	5.36	NM_014225.5
4	295	Head and neck	AG1800296FFP	3.06	MDM4	CN_Amp	NA	3.55	NM_002393.4
4	295	Head and neck	AG1800296FFP	3.06	GNAS	Missense_Mutation	p.P207L	24.89	NM_080425.3
4	295	Head and neck	AG1800296FFP	3.06	PRKDC	CN_Amp	NA	6.85	NM_006904.6

4	295	Head and neck	AG1800296FFP	3.06	NBN	CN_Amp	NA	6.56	NM_002485.4
4	295	Head and neck	AG1800296FFP	3.06	RUNX1T1	CN_Amp	NA	6.04	NM_001198679.1
4	295	Head and neck	AG1800296FFP	3.06	MYC	CN_Amp	NA	6.08	NM_002467.4
5	295	Head and neck	AG1900500FFP	1.02	ATM	Missense_Mutation	p.A2346V	46.96	NM_000051.3
5	295	Head and neck	AG1900500FFP	1.02	EMSY	CN_Amp	NA	5.89	NM_001300942.1
5	295	Head and neck	AG1900500FFP	1.02	MDM4	CN_Amp	NA	3.27	NM_002393.4
5	295	Head and neck	AG1900500FFP	1.02	CCND3	CN_Amp	NA	3.36	NM_001760.4
5	295	Head and neck	AG1900500FFP	1.02	FANCE	CN_Amp	NA	3.69	NM_021922.2
6	295	Head and neck	AG1801060FFP	21.43	MCL1	Missense_Mutation	p.I237V	21.13	NM_021960.4
6	295	Head and neck	AG1801060FFP	21.43	SPEN	Missense_Mutation	p.Y3306C	29.24	NM_015001.2
6	295	Head and neck	AG1801060FFP	21.43	ERBB3	Missense_Mutation	p.G561D	25.08	NM_001982.3
6	295	Head and neck	AG1801060FFP	21.43	BCL2L2	Missense_Mutation	p.T175M	23.16	NM_001199839.1
6	295	Head and neck	AG1801060FFP	21.43	CREBBP	Splice_Site	c.3370-7_3370-4del	16.77	NM_004380.2
6	295	Head and neck	AG1801060FFP	21.43	MAP2K4	Missense_Mutation	p.T109A	26.89	NM_001281435.1
6	295	Head and neck	AG1801060FFP	21.43	NF1	Frame_Shift	p.Y580fs	21.42	NM_000267.3
6	295	Head and neck	AG1801060FFP	21.43	NF1	Frame_Shift	p.F945fs	29.12	NM_000267.3
6	295	Head and neck	AG1801060FFP	21.43	CDK12	Frame_Shift	p.T1463fs	34.76	NM_016507.3
6	295	Head and neck	AG1801060FFP	21.43	SMARCA4	Missense_Mutation	p.A26V	6.40	NM_001128849.1
6	295	Head and neck	AG1801060FFP	21.43	CIC	Splice_Site	c.1360+4A>G	15.74	NM_015125.4
6	295	Head and neck	AG1801060FFP	21.43	EP300	Splice_Site	c.2242-6_2242-4del	28.60	NM_001429.3
6	295	Head and neck	AG1801060FFP	21.43	PBRM1	Missense_Mutation	p.C1010R	41.56	NM_018313.4
6	295	Head and neck	AG1801060FFP	21.43	PBRM1	Missense_Mutation	p.E535A	9.92	NM_018313.4
6	295	Head and neck	AG1801060FFP	21.43	DAXX	Missense_Mutation	p.R246H	22.51	NM_001141970.1
6	295	Head and neck	AG1801060FFP	21.43	FANCE	Splice_Site	p.Q323R	15.51	NM_021922.2
6	295	Head and neck	AG1801060FFP	21.43	PNRC1	Nonsense_Mutation	p.S184*	26.02	NM_006813.2
6	295	Head and neck	AG1801060FFP	21.43	EGFR	Missense_Mutation	p.M947T	14.82	NM_005228.3
6	295	Head and neck	AG1801060FFP	21.43	TRRAP	Frame_Shift	p.G1160fs	21.13	NM_001244580.1
6	295	Head and neck	AG1801060FFP	21.43	PRKDC	Nonsense_Mutation	p.R1184*	20.18	NM_006904.6
6	295	Head and neck	AG1801060FFP	21.43	JAK2	Missense_Mutation	p.M535V	20.05	NM_004972.3
7	295	Head and neck	AG1800005FFP	3.06	LRP1B	Frame_Shift	p.Q2844fs	28.00	NM_018557.2
7	295	Head and neck	AG1800005FFP	3.06	NOTCH2	CN_Amp	NA	13.26	NM_024408.3
7	295	Head and neck	AG1800005FFP	3.06	FGFR3	CN_Amp	NA	4.06	NM_000142.4
7	295	Head and neck	AG1800005FFP	3.06	KIT	Missense_Mutation	p.W557S	43.90	NM_000222.2
7	295	Head and neck	AG1800005FFP	3.06	FANCE	CN_Amp	NA	4.42	NM_021922.2
7	295	Head and neck	AG1800005FFP	3.06	DAXX	CN_Amp	NA	4.76	NM_001141970.1
7	295	Head and neck	AG1800005FFP	3.06	NOTCH4	CN_Amp	NA	4.13	NM_004557.3
7	295	Head and neck	AG1800005FFP	3.06	IRF4	CN_Amp	NA	4.62	NM_002460.3
7	295	Head and neck	AG1800005FFP	3.06	TP53	Missense_Mutation	p.P151H	20.20	NM_000546.5
7	295	Head and neck	AG1800005FFP	3.06	CDK6	CN_Amp	NA	3.29	NM_001145306.1

8	295	Head and neck	AG1700497FFP	5.10	NRAS	Missense_Mutation	p.G12V	11.04	NM_002524.4
8	295	Head and neck	AG1700497FFP	5.10	ROS1	Missense_Mutation	p.I1685L	30.40	NM_002944.2
8	295	Head and neck	AG1700497FFP	5.10	TSC1	Missense_Mutation	p.S427T	16.30	NM_000368.4
8	295	Head and neck	AG1700497FFP	5.10	BRAF	Missense_Mutation	p.D594G	12.90	NM_004333.4
8	295	Head and neck	AG1700497FFP	5.10	BCL6	Missense_Mutation	p.S186G	30.80	NM_001130845.1
9	295	Head and neck	AG1801025FFP	17.35	JAK3	CN_Amp	NA	3.74	NM_000215.3
9	295	Head and neck	AG1801025FFP	17.35	PIK3R2	CN_Amp	NA	5.08	NM_005027.3
9	295	Head and neck	AG1801025FFP	17.35	STK11	CN_Amp	NA	5.69	NM_000455.4
9	295	Head and neck	AG1801025FFP	17.35	DOT1L	CN_Amp	NA	4.38	NM_032482.2
9	295	Head and neck	AG1801025FFP	17.35	GNA11	CN_Amp	NA	4.21	NM_002067.4
9	295	Head and neck	AG1801025FFP	17.35	ETV5	CN_Amp	NA	3.69	NM_004454.2
9	295	Head and neck	AG1801025FFP	17.35	CCND3	CN_Amp	NA	4.64	NM_001760.4
9	295	Head and neck	AG1801025FFP	17.35	FANCE	CN_Amp	NA	3.85	NM_021922.2
9	295	Head and neck	AG1801025FFP	17.35	NOTCH4	CN_Amp	NA	3.46	NM_004557.3
9	295	Head and neck	AG1801025FFP	17.35	DAXX	CN_Amp	NA	7.99	NM_001141970.1
9	295	Head and neck	AG1801025FFP	17.35	HLA-A	CN_Amp	NA	4.53	NM_001242758.1.7
9	295	Head and neck	AG1801025FFP	17.35	IRF4	CN_Amp	NA	7.61	NM_002460.3
9	295	Head and neck	AG1801025FFP	17.35	CDK6	CN_Amp	NA	5.06	NM_001145306.1
9	295	Head and neck	AG1801025FFP	17.35	MET	CN_Amp	NA	4.25	NM_000245.3
9	295	Head and neck	AG1801025FFP	17.35	MYC	CN_Amp	NA	4.69	NM_002467.4
9	295	Head and neck	AG1801025FFP	17.35	TSC1	CN_Amp	NA	3.54	NM_000368.4
9	295	Head and neck	AG1801025FFP	17.35	NOTCH1	CN_Amp	NA	4.57	NM_017617.4
10	295	Head and neck	AG1800936FFP	3.06	FAT3	Missense_Mutation	p.I256N	23.04	NM_001008781.2
10	295	Head and neck	AG1800936FFP	3.06	CCND1	CN_Amp	NA	4.59	NM_053056.2
10	295	Head and neck	AG1800936FFP	3.06	EMSY	CN_Amp	NA	6.55	NM_001300942.1
10	295	Head and neck	AG1800936FFP	3.06	FLT3	CN_Amp	NA	4.80	NM_004119.2
10	295	Head and neck	AG1800936FFP	3.06	FLT1	CN_Amp	NA	6.38	NM_002019.4
10	295	Head and neck	AG1800936FFP	3.06	TSHR	Missense_Mutation	p.M396I	14.29	NM_000369.2
10	295	Head and neck	AG1800936FFP	3.06	MAP2K4	CN_Amp	NA	3.66	NM_001281435.1
10	295	Head and neck	AG1800936FFP	3.06	RAD51C	CN_Amp	NA	3.71	NM_058216.2
10	295	Head and neck	AG1800936FFP	3.06	BRIP1	CN_Amp	NA	4.89	NM_032043.2
10	295	Head and neck	AG1800936FFP	3.06	SMARCA4	CN_Amp	NA	4.61	NM_001128849.1
10	295	Head and neck	AG1800936FFP	3.06	MDM4	CN_Amp	NA	3.70	NM_002393.4
10	295	Head and neck	AG1800936FFP	3.06	FBXW7	CN_Amp	NA	3.94	NM_033632.3
10	295	Head and neck	AG1800936FFP	3.06	HGF	Missense_Mutation	p.R702C	40.35	NM_000601.5
10	295	Head and neck	AG1800936FFP	3.06	ETV1	CN_Amp	NA	4.66	NM_004956.4
10	295	Head and neck	AG1800936FFP	3.06	PMS2	CN_Amp	NA	4.09	NM_000535.6
10	295	Head and neck	AG1800936FFP	3.06	CARD11	CN_Amp	NA	4.08	NM_032415.5
10	295	Head and neck	AG1800936FFP	3.06	NBN	CN_Amp	NA	5.73	NM_002485.4

10	295	Head and neck	AG1800936FFP	3.06	RUNX1T1	CN_Amp	NA	5.59	NM_001198679.1
10	295	Head and neck	AG1800936FFP	3.06	MYC	CN_Amp	NA	5.98	NM_002467.4
11	1021	Head and neck	2103166256	3.84	CTNNB1	Missense_Mutation	p.S33P	0.50	NM_001904.3
11	1021	Head and neck	2103166256	3.84	MST1R	Missense_Mutation	p.R142H	0.46	NM_002447.2
11	1021	Head and neck	2103166256	3.84	FGFR1	Missense_Mutation	p.A21T	0.27	NM_023110.2
11	1021	Head and neck	2103166256	3.84	BLM	Missense_Mutation	p.D219N	16.40	NM_000057.2
12	1021	Head and neck	2005283981	2.88	ZBTB16	Missense_Mutation	p.Y297C	21.80	NM_006006.4
12	1021	Head and neck	2005283981	2.88	MLH3	Missense_Mutation	p.G1396R	21.70	NM_001040108.1
12	1021	Head and neck	2005283981	2.88	EP300	Missense_Mutation	p.Q570L	6.20	NM_001429.3
13	1021	Head and neck	1911137249	13.46	EPHA2	Missense_Mutation	p.R861H	16.70	NM_004431.3
13	1021	Head and neck	1911137249	13.46	ALK	Missense_Mutation	p.V623L	16.20	NM_004304.4
13	1021	Head and neck	1911137249	13.46	VHL	Missense_Mutation	p.E46G	3.10	NM_000551.3
13	1021	Head and neck	1911137249	13.46	FAT1	Missense_Mutation	p.F2809I	12.50	NM_005245.3
13	1021	Head and neck	1911137249	13.46	TERT	5'UTR	c.146C>T	35.40	NM_198253.2
13	1021	Head and neck	1911137249	13.46	FOXQ1	Missense_Mutation	p.I132V	5.80	NM_033260.3
13	1021	Head and neck	1911137249	13.46	WRN	In_Frame	p.E510del	5.30	NM_000553.4
13	1021	Head and neck	1911137249	13.46	FANCC	Missense_Mutation	p.V323A	3.40	NM_000136.2
13	1021	Head and neck	1911137249	13.46	CBL	Splice_Site	c.1096-1G>C	50.20	NM_005188.3
13	1021	Head and neck	1911137249	13.46	ARID2	Missense_Mutation	p.I481V	5.00	NM_152641.2
13	1021	Head and neck	1911137249	13.46	MLH3	Missense_Mutation	p.S184F	19.20	NM_001040108.1
13	1021	Head and neck	1911137249	13.46	SMAD3	Missense_Mutation	p.Q222E	14.10	NM_005902.3
13	1021	Head and neck	1911137249	13.46	TSHZ2	Nonsense_Mutation	p.Q668*	37.70	NM_173485.5
13	1021	Head and neck	1911137249	13.46	BCOR	Missense_Mutation	p.V806A	23.10	NM_001123385.1
13	1021	Head and neck	1911137249	13.46	MDM2	CN_Amp	NA	13.33	NM_002392.4
13	1021	Head and neck	1911137249	13.46	NBN	CN_Amp	NA	5.76	NM_002485.4
14	1021	Head and neck	2005143432	6.73	BRAF	Missense_Mutation	p.V600E	46.30	NM_004333.4
14	1021	Head and neck	2005143432	6.73	IKZF1	Missense_Mutation	p.D28E	40.50	NM_006060.4
14	1021	Head and neck	2005143432	6.73	AKT2	Missense_Mutation	p.R253W	28.50	NM_001626.4
14	1021	Head and neck	2005143432	6.73	ERBB4	Missense_Mutation	p.K1223T	18.20	NM_005235.2
14	1021	Head and neck	2005143432	6.73	GRM3	Missense_Mutation	p.L741F	14.50	NM_000840.2
14	1021	Head and neck	2005143432	6.73	KDM5A	Missense_Mutation	p.R785S	10.20	NM_001042603.1
14	1021	Head and neck	2005143432	6.73	IRF4	Missense_Mutation	p.L307V	10.00	NM_002460.3
15	1021	Head and neck	2006154689	2.88	ROBO2	Missense_Mutation	p.A70V	4.90	NM_001128929.2
15	1021	Head and neck	2006154689	2.88	COPS2	Missense_Mutation	p.L195P	32.90	NM_001143887.1
15	1021	Head and neck	2006154689	2.88	NF1	Frame_Shift	p.D888Afs*10	29.20	NM_000267.3
15	1021	Head and neck	2006154689	2.88	CCND1	CN_Amp	NA	20.54	NM_053056.2
15	1021	Head and neck	2006154689	2.88	FGF19	CN_Amp	NA	10.21	NM_005117.2
15	1021	Head and neck	2006154689	2.88	FGF4	CN_Amp	NA	10.14	NM_002007.2
15	1021	Head and neck	2006154689	2.88	FGF3	CN_Amp	NA	8.90	NM_005247.2

16	1021	Head and neck	1908153704	5.77	NRAS	Missense_Mutation	p.G12C	97.40	NM_002524.4
16	1021	Head and neck	1908153704	5.77	ZFPM2	Missense_Mutation	p.P387S	11.80	NM_012082.3
16	1021	Head and neck	1908153704	5.77	FGFR2	Missense_Mutation	p.G182W	49.60	NM_000141.4
16	1021	Head and neck	1908153704	5.77	CBL	Missense_Mutation	p.S439N	44.10	NM_005188.3
16	1021	Head and neck	1908153704	5.77	MLL2	Missense_Mutation	p.E4939K	48.90	NM_003482.3
16	1021	Head and neck	1908153704	5.77	AURKB	Nonsense_Mutation	p.Q112*	51.40	NM_004217.3
16	1021	Head and neck	1908153704	5.77	NOTCH2	CN_Amp	NA	6.40	NM_024408.3
16	1021	Head and neck	1908153704	5.77	CDKN1A	CN_Amp	NA	6.71	NM_078467.2
16	1021	Head and neck	1908153704	5.77	FGFR1	CN_Amp	NA	4.06	NM_023110.2
16	1021	Head and neck	1908153704	5.77	NBN	CN_Amp	NA	14.90	NM_002485.4
16	1021	Head and neck	1908153704	5.77	MYC	CN_Amp	NA	11.52	NM_002467.4
17	295	Head and neck	AG1700419FFP	7.14	BRAF	Missense_Mutation	p.V600E	40.20	NM_004333.4
17	295	Head and neck	AG1700419FFP	7.14	BLM	Missense_Mutation	p.R859K	45.80	NM_000057.3
17	295	Head and neck	AG1700419FFP	7.14	TP53	Missense_Mutation	p.F113V	75.80	NM_000546.5
17	295	Head and neck	AG1700419FFP	7.14	SETD2	Missense_Mutation	p.K352E	12.00	NM_014159.6
17	295	Head and neck	AG1700419FFP	7.14	BAP1	Missense_Mutation	p.P135L	83.80	NM_004656.3
17	295	Head and neck	AG1700419FFP	7.14	NOTCH4	Missense_Mutation	p.D272G	32.90	NM_004557.3
17	295	Head and neck	AG1700419FFP	7.14	CARD11	In_Frame	p.S622del	2.80	NM_032415.5
17	295	Head and neck	AG1700419FFP	7.14	FANCF	CN_Amp	NA	7.11	NM_022725.3
17	295	Head and neck	AG1700419FFP	7.14	MYC	CN_Amp	NA	5.78	NM_002467.4
17	295	Head and neck	AG1700419FFP	7.14	SPEN	CN_Amp	NA	5.49	NM_015001.2
17	295	Head and neck	AG1700419FFP	7.14	TET2	CN_Amp	NA	4.36	NM_001127208.2
18	295	Head and neck	AG1801043FFP	3.06	ATM	Missense_Mutation	p.I2702V	27.79	NM_000051.3
18	295	Head and neck	AG1801043FFP	3.06	CCND1	CN_Amp	NA	5.32	NM_053056.2
18	295	Head and neck	AG1801043FFP	3.06	FGF19	CN_Amp	NA	3.42	NM_005117.2
18	295	Head and neck	AG1801043FFP	3.06	EMSY	CN_Amp	NA	3.67	NM_001300942.1
18	295	Head and neck	AG1801043FFP	3.06	PRKAR1A	CN_Amp	NA	4.31	NM_002734.4
18	295	Head and neck	AG1801043FFP	3.06	PDGFRA	CN_Amp	NA	5.35	NM_006206.4
18	295	Head and neck	AG1801043FFP	3.06	KIT	CN_Amp	NA	5.95	NM_000222.2
18	295	Head and neck	AG1801043FFP	3.06	KDR	CN_Amp	NA	4.38	NM_002253.2
18	295	Head and neck	AG1801043FFP	3.06	APC	Missense_Mutation	p.W699L	17.24	NM_000038.5
18	295	Head and neck	AG1801043FFP	3.06	NOTCH4	Missense_Mutation	p.G1622V	5.90	NM_004557.3
18	295	Head and neck	AG1801043FFP	3.06	DAXX	CN_Amp	NA	4.50	NM_001141970.1
19	295	Head and neck	AG1800507FFP	4.08	CDK8	CN_Amp	NA	3.79	NM_001260.2
19	295	Head and neck	AG1800507FFP	4.08	BRCA2	CN_Amp	NA	3.63	NM_000059.3
19	295	Head and neck	AG1800507FFP	4.08	DIS3	CN_Amp	NA	3.51	NM_014953.4
19	295	Head and neck	AG1800507FFP	4.08	JAK3	Missense_Mutation	p.E331K	25.76	NM_000215.3
19	295	Head and neck	AG1800507FFP	4.08	BRAF	Missense_Mutation	p.V600E	35.23	NM_004333.4
19	295	Head and neck	AG1800507FFP	4.08	ADGRA2	Missense_Mutation	p.R255C	32.88	NM_032777.9

19	295	Head and neck	AG1800507FFP	4.08	FGFR1	CN_Del	NA	0.94	NM_023110.2
19	295	Head and neck	AG1800507FFP	4.08	CDKN2A	In_Frame	p.L32_L37del	46.39	NM_000077.4
19	295	Head and neck	AG1800507FFP	4.08	NOTCH1	CN_Amp	NA	3.72	NM_017617.4
20	295	Head and neck	AG1800198FFP	2.04	NRAS	Missense_Mutation	p.Q61R	40.44	NM_002524.4
20	295	Head and neck	AG1800198FFP	2.04	CARD11	Splice_Site	p.Q381=	42.32	NM_032415.5
20	295	Head and neck	AG1800198FFP	2.04	KAT6A	CN_Del	NA	1.04	NM_006766.4
20	295	Head and neck	AG1800198FFP	2.04	FGFR1	CN_Del	NA	0.96	NM_023110.2
20	295	Head and neck	AG1800198FFP	2.04	PRKDC	CN_Amp	NA	4.38	NM_006904.6
20	295	Head and neck	AG1800198FFP	2.04	NBN	CN_Amp	NA	5.58	NM_002485.4
20	295	Head and neck	AG1800198FFP	2.04	MYC	CN_Amp	NA	6.74	NM_002467.4
20	295	Head and neck	AG1800198FFP	2.04	PAX5	CN_Del	NA	0.93	NM_016734.2
20	295	Head and neck	AG1800198FFP	2.04	FANCG	CN_Del	NA	1.03	NM_004629.1
21	295	Head and neck	AG1901111FFP	6.12	SUFU	Missense_Mutation	p.P24T	15.43	NM_016169.3
21	295	Head and neck	AG1901111FFP	6.12	ARID2	Missense_Mutation	p.K35Q	40.33	NM_152641.2
21	295	Head and neck	AG1901111FFP	6.12	IRS2	Missense_Mutation	p.E878D	61.04	NM_003749.2
21	295	Head and neck	AG1901111FFP	6.12	CTNNB1	Missense_Mutation	p.S318G	40.22	NM_001904.3
21	295	Head and neck	AG1901111FFP	6.12	TET2	Missense_Mutation	p.L738P	26.48	NM_001127208.2
21	295	Head and neck	AG1901111FFP	6.12	FGFR3	CN_Amp	NA	6.85	NM_000142.4
21	295	Head and neck	AG1901111FFP	6.12	NOTCH1	CN_Amp	NA	4.15	NM_017617.4
21	295	Head and neck	AG1901111FFP	6.12	KDM5C	Missense_Mutation	p.E1432V	78.23	NM_004187.3
22	295	Uvea	AG1700498FFP	5.10	NOTCH1	Missense_Mutation	p.V1754M	45.50	NM_017617.4
22	295	Uvea	AG1700498FFP	5.10	SPEN	Missense_Mutation	p.G1659D	74.90	NM_015001.2
22	295	Uvea	AG1700498FFP	5.10	AXL	Missense_Mutation	p.P422S	50.20	NM_021913.4
22	295	Uvea	AG1700498FFP	5.10	EGFR	Missense_Mutation	p.V786M	51.04	NM_005228.3
22	295	Uvea	AG1700498FFP	5.10	GNAQ	Missense_Mutation	p.Q209L	32.80	NM_002072.4
23	295	Uvea	AG1900949FFP	3.06	TSC2	Splice_Site	p.L741F	51.25	NM_000548.4
23	295	Uvea	AG1900949FFP	3.06	BRCA1	Frame_Shift	p.A322fs	46.50	NM_007294.3
23	295	Uvea	AG1900949FFP	3.06	GNAS	Missense_Mutation	p.D452N	9.14	NM_080425.3
25	295	Uvea	AG1901088FFP	1.02	DAXX	CN_Amp	NA	4.10	NM_001141970.1
25	295	Uvea	AG1901088FFP	1.02	GNAQ	Missense_Mutation	p.Q209L	22.31	NM_002072.4
26	295	Uvea	AG1800923FFP	7.14	GNAQ	Missense_Mutation	p.Q209P	39.29	NM_002072.4
26	295	Uvea	AG1800923FFP	7.14	SPEN	Nonsense_Mutation	p.E2125*	68.69	NM_015001.2
26	295	Uvea	AG1800923FFP	7.14	FAT3	Nonsense_Mutation	p.Y48*	42.04	NM_001008781.2
26	295	Uvea	AG1800923FFP	7.14	FAT3	Missense_Mutation	p.T60A	44.27	NM_001008781.2
26	295	Uvea	AG1800923FFP	7.14	PARP4	Missense_Mutation	p.L1592P	43.43	NM_006437.3
26	295	Uvea	AG1800923FFP	7.14	FBXW7	Splice_Site	c.1237-2A>G	63.79	NM_033632.3
26	295	Uvea	AG1800923FFP	7.14	PDGFRB	Missense_Mutation	p.R115W	36.52	NM_002609.3
26	295	Uvea	AG1800923FFP	7.14	MYC	CN_Amp	NA	4.75	NM_002467.4
27	1021	Uvea	1910145972	1.92	GNA11	Missense_Mutation	p.Q209L	20.60	NM_002067.2

27	1021	Uvea	1910145972	1.92	CDK12	Missense_Mutation	p.A361P	4.30	NM_016507.2
28	295	Head and neck	AG1800811FFP	5.10	ARID1A	Frame_Shift	p.V1185fs	14.76	NM_006015.4
28	295	Head and neck	AG1800811FFP	5.10	TP53	Nonsense_Mutation	p.R306*	24.73	NM_000546.5
28	295	Head and neck	AG1800811FFP	5.10	LRP1B	Missense_Mutation	p.D1081Y	21.11	NM_018557.2
28	295	Head and neck	AG1800811FFP	5.10	ATR	Missense_Mutation	p.E54K	23.67	NM_001184.3
28	295	Head and neck	AG1800811FFP	5.10	PTCH1	Frame_Shift	p.L1048fs	15.18	NM_000264.3
29	1021	Head and neck	2009168987	1.92	BRAF	Missense_Mutation	p.D594N	8.00	NM_004333.4
29	1021	Head and neck	2009168987	1.92	BRCA2	Missense_Mutation	p.K123N	7.00	NM_000059.3
30	1021	Head and neck	2001200259	4.81	NOTCH4	Missense_Mutation	p.P1911R	27.50	NM_004557.3
30	1021	Head and neck	2001200259	4.81	RUNX1	Frame_Shift	p.A470Lfs*131	29.70	NM_001754.4
30	1021	Head and neck	2001200259	4.81	PTPRD	Missense_Mutation	p.T277K	11.70	NM_002839.3
30	1021	Head and neck	2001200259	4.81	DDR1	Nonsense_Mutation	p.W258*	8.20	NM_001954.4
30	1021	Head and neck	2001200259	4.81	BRD4	Missense_Mutation	p.K378E	2.00	NM_058243.2
31	295	Head and neck	AG1801089FFP	1.02	NRAS	Missense_Mutation	p.Q61R	9.90	NM_002524.4
32	1021	Head and neck	2005294059	4.81	CDKN2A	Frame_Shift	p.A21Gfs*23	77.50	NM_000077.4
32	1021	Head and neck	2005294059	4.81	PTPN11	Missense_Mutation	p.T507K	90.20	NM_002834.3
32	1021	Head and neck	2005294059	4.81	PAX5	Frame_Shift	p.A322Rfs*19	38.10	NM_016734.1
32	1021	Head and neck	2005294059	4.81	SERPINB3	Missense_Mutation	p.H330R	28.40	NM_006919.2
32	1021	Head and neck	2005294059	4.81	NOTCH3	Missense_Mutation	p.R244Q	21.20	NM_000435.2
34	295	Head and neck	AG1801063FFP	3.06	NF1	Nonsense_Mutation	p.S1100*	38.91	NM_000267.3
34	295	Head and neck	AG1801063FFP	3.06	FBXW7	Nonsense_Mutation	p.R278*	41.80	NM_033632.3
34	295	Head and neck	AG1801063FFP	3.06	EGFR	Missense_Mutation	p.T693R	37.08	NM_005228.3
34	295	Head and neck	AG1801063FFP	3.06	PTEN	CN_Del	NA	0.47	NM_000314.6
34	295	Head and neck	AG1801063FFP	3.06	RET	CN_Del	NA	0.97	NM_020975.4
34	295	Head and neck	AG1801063FFP	3.06	DOT1L	CN_Amp	NA	3.57	NM_032482.2
34	295	Head and neck	AG1801063FFP	3.06	GNA11	CN_Amp	NA	4.91	NM_002067.4
35	295	Head and neck	AG1800013FFP	10.20	NF1	Splice_Site	p.G96G	80.60	NM_000267.3
35	295	Head and neck	AG1800013FFP	10.20	KCTD15-BRAF	Fusion	NA	33.50	NM_024076.3;NM_004333.4
35	295	Head and neck	AG1800013FFP	10.20	KMT2A	Frame_Shift	p.R1487fs	76.70	NM_001197104.1
35	295	Head and neck	AG1800013FFP	10.20	KMT2D	Missense_Mutation	p.M1660V	52.60	NM_003482.3
35	295	Head and neck	AG1800013FFP	10.20	PIK3C2G	Missense_Mutation	p.D1048N	51.30	NM_001288772.1
35	295	Head and neck	AG1800013FFP	10.20	ASXL1	Missense_Mutation	p.Q708E	42.60	NM_015338.5
35	295	Head and neck	AG1800013FFP	10.20	CCND1	CN_Amp	NA	6.87	NM_053056.2
35	295	Head and neck	AG1800013FFP	10.20	FGF19	CN_Amp	NA	5.39	NM_005117.2
35	295	Head and neck	AG1800013FFP	10.20	FGF3	CN_Amp	NA	4.86	NM_005247.2
35	295	Head and neck	AG1800013FFP	10.20	FGF4	CN_Amp	NA	5.83	NM_002007.2
35	295	Head and neck	AG1800013FFP	10.20	EMSY	CN_Amp	NA	8.64	NM_001300942.1
35	295	Head and neck	AG1800013FFP	10.20	MRE11A	CN_Amp	NA	4.02	NM_005591.3

35	295	Head and neck	AG1800013FFP	10.20	LRP1B	Missense_Mutation	p.D1279G	3.70	NM_018557.2
35	295	Head and neck	AG1800013FFP	10.20	LRP1B	Splice_Site	NA	24.70	NM_018557.2
35	295	Head and neck	AG1800013FFP	10.20	CCNE1	CN_Amp	NA	3.55	NM_001238.3
35	295	Head and neck	AG1800013FFP	10.20	AKT2	CN_Amp	NA	4.04	NM_001626.4
35	295	Head and neck	AG1800013FFP	10.20	MITF	CN_Amp	NA	3.84	NM_000248.3
35	295	Head and neck	AG1800013FFP	10.20	CTNNB1	CN_Amp	NA	3.71	NM_001904.3
35	295	Head and neck	AG1800013FFP	10.20	CTNNB1	Missense_Mutation	p.T41A	17.00	NM_001904.3
35	295	Head and neck	AG1800013FFP	10.20	ETV1	CN_Amp	NA	3.55	NM_004956.4
35	295	Head and neck	AG1800013FFP	10.20	NTRK3	Missense_Mutation	p.I823T	15.20	NM_001012338.2
35	295	Head and neck	AG1800013FFP	10.20	EPHA3	Missense_Mutation	p.L455W	31.40	NM_005233.5
36	295	Head and neck	AG1900087FFP	1.02	BRAF	Missense_Mutation	p.G466A	10.80	NM_004333.4
36	295	Head and neck	AG1900087FFP	1.02	WDR64-AKT3	Fusion	NA	27.65	NM_001367482.1; NM_005465.4
36	295	Head and neck	AG1900087FFP	1.02	CDK4	CN_Amp	NA	6.56	NM_000075.3
36	295	Head and neck	AG1900087FFP	1.02	MDM2	CN_Amp	NA	8.54	NM_002392.5
37	295	Head and neck	AG1900237FFP	2.04	BRAF	Missense_Mutation	p.S339P	25.74	NM_004333.4
37	295	Head and neck	AG1900237FFP	2.04	PTCH1	Missense_Mutation	p.N555Y	22.19	NM_000264.3
37	295	Head and neck	AG1900237FFP	2.04	CDK4	CN_Amp	NA	4.49	NM_000075.3
37	295	Head and neck	AG1900237FFP	2.04	MDM2	CN_Amp	NA	3.91	NM_002392.5
37	295	Head and neck	AG1900237FFP	2.04	ARID2	CN_Amp	NA	3.73	NM_152641.2
37	295	Head and neck	AG1900237FFP	2.04	PTPN11	CN_Amp	NA	4.16	NM_002834.3
38	1021	Head and neck	2011030955	5.77	ATRX	Nonsense_Mutation	p.K994*	80.70	NM_000489.3
38	1021	Head and neck	2011030955	5.77	NRAS	Missense_Mutation	p.Q61K	60.50	NM_002524.4
38	1021	Head and neck	2011030955	5.77	ASPM	Missense_Mutation	p.K485R	20.30	NM_018136.4
38	1021	Head and neck	2011030955	5.77	EPHA3	Missense_Mutation	p.F801S	15.70	NM_005233.5
38	1021	Head and neck	2011030955	5.77	BCL9	Missense_Mutation	p.P1023A	14.40	NM_004326.2
38	1021	Head and neck	2011030955	5.77	NOTCH3	Missense_Mutation	p.R2022H	2.80	NM_000435.2
38	1021	Head and neck	2011030955	5.77	MDM2	CN_Amp	NA	6.40	NM_002392.4
38	1021	Head and neck	2011030955	5.77	CDK4	CN_Amp	NA	6.00	NM_000075.3
38	1021	Head and neck	2011030955	5.77	FOXA1	CN_Amp	NA	5.40	NM_004496.3
38	1021	Head and neck	2011030955	5.77	RAD51B	CN_Amp	NA	5.20	NM_133509.3
38	1021	Head and neck	2011030955	5.77	NKX2-1	CN_Amp	NA	5.00	NM_001079668.2
38	1021	Head and neck	2011030955	5.77	HNF1A	CN_Amp	NA	4.60	NM_000545.5
38	1021	Head and neck	2011030955	5.77	FANCM	CN_Amp	NA	4.60	NM_020937.2
38	1021	Head and neck	2011030955	5.77	MAX	CN_Amp	NA	4.40	NM_002382.4
39	295	Head and neck	AG1801211FFP	6.12	KIT	Missense_Mutation	p.I319V	84.42	NM_000222.2
39	295	Head and neck	AG1801211FFP	6.12	KIT	Missense_Mutation	p.Y553C	84.40	NM_000222.2
39	295	Head and neck	AG1801211FFP	6.12	CIC	Missense_Mutation	p.P911Q	59.97	NM_015125.4
39	295	Head and neck	AG1801211FFP	6.12	BRCA1	Missense_Mutation	p.T796I	44.43	NM_007294.3

39	295	Head and neck	AG1801211FFP	6.12	FAT3	Missense_Mutation	p.Y941C	39.12	NM_001008781.2
39	295	Head and neck	AG1801211FFP	6.12	TERT	5'UTR	c.146C>T	NA	NM_198253.2
39	295	Head and neck	AG1801211FFP	6.12	CDK4	CN_Amp	NA	13.66	NM_000075.3
39	295	Head and neck	AG1801211FFP	6.12	KMT2D	CN_Amp	NA	3.44	NM_003482.3
39	295	Head and neck	AG1801211FFP	6.12	FANCL	CN_Amp	NA	4.37	NM_018062.3
39	295	Head and neck	AG1801211FFP	6.12	PDGFRA	CN_Amp	NA	2.97	NM_006206.4
39	295	Head and neck	AG1801211FFP	6.12	KIT	CN_Amp	NA	4.94	NM_000222.2
39	295	Head and neck	AG1801211FFP	6.12	KDR	CN_Amp	NA	3.75	NM_002253.2
40	1021	Head and neck	2006235102	7.69	BRAF	Missense_Mutation	p.V600E	61.00	NM_004333.4
40	1021	Head and neck	2006235102	7.69	TERT	5'UTR	c.146C>T	30.30	NM_198253.2
40	1021	Head and neck	2006235102	7.69	DNAH6	Missense_Mutation	p.P641L	11.90	NM_001370.1
40	1021	Head and neck	2006235102	7.69	TGFBR1	Missense_Mutation	p.S57F	15.80	NM_004612.2
40	1021	Head and neck	2006235102	7.69	CBL	Missense_Mutation	p.P453S	28.40	NM_005188.3
40	1021	Head and neck	2006235102	7.69	MLL2	Missense_Mutation	p.P496L	30.00	NM_003482.3
40	1021	Head and neck	2006235102	7.69	ERBB4	Nonsense_Mutation	p.E563*	1.00	NM_005235.2
40	1021	Head and neck	2006235102	7.69	EP300	Missense_Mutation	p.R397G	28.50	NM_001429.3
40	1021	Head and neck	2006235102	7.69	BRCA2	CN_Amp	NA	5.17	NM_000059.3
41	295	Head and neck	AG1800405FFP	7.14	BRAF	Missense_Mutation	p.V600E	18.58	NM_004333.4
41	295	Head and neck	AG1800405FFP	7.14	ARID1A	Frame_Shift	p.R1980fs	12.30	NM_006015.4
41	295	Head and neck	AG1800405FFP	7.14	FAT3	Missense_Mutation	p.R2010H	53.65	NM_001008781.2
41	295	Head and neck	AG1800405FFP	7.14	ASXL1	Missense_Mutation	p.K1174E	17.45	NM_015338.5
41	295	Head and neck	AG1800405FFP	7.14	TET2	Frame_Shift	p.S1559fs	51.30	NM_001127208.2
41	295	Head and neck	AG1800405FFP	7.14	FBXW7	Missense_Mutation	p.G391A	20.71	NM_033632.3
41	295	Head and neck	AG1800405FFP	7.14	TERT	5'UTR	c.146C>T	NA	NM_198253.2
42	295	Head and neck	AG1901003FFP	6.12	NF1	Frame_Shift	p.P931fs	70.56	NM_000267.3
42	295	Head and neck	AG1901003FFP	6.12	IGF1R	Missense_Mutation	p.H959P	31.10	NM_000875.4
42	295	Head and neck	AG1901003FFP	6.12	CBL	Missense_Mutation	p.P548S	15.41	NM_005188.3
42	295	Head and neck	AG1901003FFP	6.12	FAT3	Missense_Mutation	p.D1878N	32.90	NM_001008781.2
42	295	Head and neck	AG1901003FFP	6.12	TRRAP	Missense_Mutation	p.M658I	18.14	NM_001244580.1
42	295	Head and neck	AG1901003FFP	6.12	PDGFRB	Missense_Mutation	p.G965A	9.26	NM_002609.3
42	295	Head and neck	AG1901003FFP	6.12	DDR2	CN_Amp	NA	3.79	NM_001014796.1
42	295	Head and neck	AG1901003FFP	6.12	MDM4	CN_Amp	NA	3.86	NM_002393.4
42	295	Head and neck	AG1901003FFP	6.12	AKT3	CN_Amp	NA	3.64	NM_005465.4
42	295	Head and neck	AG1901003FFP	6.12	CCND3	CN_Amp	NA	4.39	NM_001760.4
42	295	Head and neck	AG1901003FFP	6.12	DAXX	CN_Amp	NA	4.06	NM_001141970.1
42	295	Head and neck	AG1901003FFP	6.12	HLA-A	CN_Amp	NA	4.93	NM_001242758.1.7
42	295	Head and neck	AG1901003FFP	6.12	IKZF1	CN_Amp	NA	6.56	NM_006060.5
42	295	Head and neck	AG1901003FFP	6.12	PRKDC	CN_Amp	NA	5.74	NM_006904.6

42	295	Head and neck	AG1901003FFP	6.12	NBN	CN_Amp	NA	6.03	NM_002485.4
42	295	Head and neck	AG1901003FFP	6.12	RUNX1T1	CN_Amp	NA	5.31	NM_001198679.1
42	295	Head and neck	AG1901003FFP	6.12	IRS2	CN_Amp	NA	4.47	NM_003749.2
43	1021	Esophagus	1911157416	1.92	PARK2	Missense_Mutation	p.V269M	29.10	NM_004562.2
43	1021	Esophagus	1911157416	1.92	FANCM	Frame_Shift	p.G1235Wfs*4	41.40	NM_020937.2
43	1021	Esophagus	1911157416	1.92	FBXW7	CN_Del	NA	0.21	NM_033632.3
43	1021	Esophagus	1911157416	1.92	FAT1	CN_Del	NA	0.19	NM_005245.3
44	1021	Esophagus	2006235088	0.00	PTEN	CN_Del	NA	0.47	NM_000314.4
44	1021	Esophagus	2006235088	0.00	IRS2	CN_Amp	NA	6.37	NM_003749.2
45	1021	Esophagus	2008147463	9.62	ERBB4	Splice_Site	c.883+1G>A	1.10	NM_005235.2
45	1021	Esophagus	2008147463	9.62	ADAM29	Missense_Mutation	p.P752R	4.60	NM_014269.4
45	1021	Esophagus	2008147463	9.62	MIOS	Missense_Mutation	p.T773A	13.20	NM_019005.3
45	1021	Esophagus	2008147463	9.62	EXT1	Missense_Mutation	p.P82S	9.30	NM_000127.2
45	1021	Esophagus	2008147463	9.62	PTPRD	Missense_Mutation	p.S1663T	48.00	NM_002839.3
45	1021	Esophagus	2008147463	9.62	CYP2C8	Frame_Shift	p.T159Pfs*19	39.00	NM_000770.3
45	1021	Esophagus	2008147463	9.62	HNF1A	Missense_Mutation	p.R200Q	15.10	NM_000545.5
45	1021	Esophagus	2008147463	9.62	TP53	Frame_Shift	p.R110Lfs*40	1.70	NM_000546.5
45	1021	Esophagus	2008147463	9.62	TOP2A	Missense_Mutation	p.E1263G	2.30	NM_001067.3
45	1021	Esophagus	2008147463	9.62	CYP2D6	Missense_Mutation	p.F120I	33.30	NM_000106.5
45	1021	Esophagus	2008147463	9.62	MDM4	CN_Amp	NA	3.88	NM_002393.4
46	1021	Esophagus	2006225054	3.85	SF3B1	Missense_Mutation	p.R625C	18.00	NM_012433.2
46	1021	Esophagus	2006225054	3.85	BRAF	Missense_Mutation	p.K601E	28.20	NM_004333.4
46	1021	Esophagus	2006225054	3.85	GRIN2A	Missense_Mutation	p.T215K	3.10	NM_001134407.1
46	1021	Esophagus	2006225054	3.85	RUNX1	Missense_Mutation	p.P301T	18.60	NM_001754.4
46	1021	Esophagus	2006225054	3.85	PDGFRA	CN_Amp	NA	12.40	NM_006206.4
46	1021	Esophagus	2006225054	3.85	KIT	CN_Amp	NA	9.48	NM_000222.2
46	1021	Esophagus	2006225054	3.85	GRM3	CN_Amp	NA	4.39	NM_000840.2
46	1021	Esophagus	2006225054	3.85	CDK6	CN_Amp	NA	3.96	NM_001145306.1
46	1021	Esophagus	2006225054	3.85	PIK3CG	CN_Amp	NA	5.26	NM_002649.2
46	1021	Esophagus	2006225054	3.85	MET	CN_Amp	NA	3.55	NM_000245.2
47	1021	Genitourinary tract	2011181604	1.92	TP53	Missense_Mutation	p.H179Q	69.90	NM_000546.5
47	1021	Genitourinary tract	2011181604	1.92	ATRX	Frame_Shift	p.K1052Nfs*2	49.70	NM_000489.3
47	1021	Genitourinary tract	2011181604	1.92	POT1	CN_Amp	NA	4.20	NM_015450.2
47	1021	Genitourinary tract	2011181604	1.92	CDK6	CN_Amp	NA	4.00	NM_001145306.1
47	1021	Genitourinary tract	2011181604	1.92	BRAF	CN_Amp	NA	3.80	NM_004333.4
47	1021	Genitourinary tract	2011181604	1.92	SMO	CN_Amp	NA	3.80	NM_005631.4
47	1021	Genitourinary tract	2011181604	1.92	MET	CN_Amp	NA	3.80	NM_000245.2
48	1021	Esophagus	2006285262	2.88	SF3B1	Missense_Mutation	p.R625C	37.50	NM_012433.2
48	1021	Esophagus	2006285262	2.88	KIT	Missense_Mutation	p.L576P	27.90	NM_000222.2

48	1021	Esophagus	2006285262	2.88	NF1	Nonsense_Mutation	p.Q2652*	25.90	NM_000267.3
48	1021	Esophagus	2006285262	2.88	DAXX	CN_Amp	NA	4.31	NM_001141970.1
48	1021	Esophagus	2006285262	2.88	BRAF	CN_Amp	NA	4.16	NM_004333.4
48	1021	Esophagus	2006285262	2.88	NBN	CN_Amp	NA	7.68	NM_002485.4
48	1021	Esophagus	2006285262	2.88	EXT1	CN_Amp	NA	4.51	NM_000127.2
48	1021	Esophagus	2006285262	2.88	MYC	CN_Amp	NA	5.88	NM_002467.4
48	1021	Esophagus	2006285262	2.88	RECQL4	CN_Amp	NA	4.53	NM_004260.3
48	1021	Esophagus	2006285262	2.88	TSC1	CN_Amp	NA	3.71	NM_000368.4
49	1021	Esophagus	2008267986	18.27	KIT	Missense_Mutation	p.L576P	53.40	NM_000222.2
49	1021	Esophagus	2008267986	18.27	PTEN	Missense_Mutation	p.S361R	67.00	NM_000314.4
49	1021	Esophagus	2008267986	18.27	ARID1A	Missense_Mutation	p.P225S	68.20	NM_006015.4
49	1021	Esophagus	2008267986	18.27	LRP1B	Missense_Mutation	p.A3191T	59.40	NM_018557.2
49	1021	Esophagus	2008267986	18.27	MST1R	Missense_Mutation	p.V366I	5.30	NM_002447.2
49	1021	Esophagus	2008267986	18.27	PIK3CA	Missense_Mutation	p.H1047R	18.40	NM_006218.2
49	1021	Esophagus	2008267986	18.27	RAD50	Missense_Mutation	p.G469E	40.40	NM_005732.3
49	1021	Esophagus	2008267986	18.27	DDR1	Missense_Mutation	p.R344C	11.00	NM_001954.4
49	1021	Esophagus	2008267986	18.27	DAXX	Missense_Mutation	p.A498G	39.20	NM_001141970.1
49	1021	Esophagus	2008267986	18.27	MLL3	Missense_Mutation	p.G908C	5.20	NM_170606.2
49	1021	Esophagus	2008267986	18.27	MYC	Missense_Mutation	p.V421L	2.60	NM_002467.4
49	1021	Esophagus	2008267986	18.27	CBL	Splice_Site	c.1096-1G>T	8.50	NM_005188.3
49	1021	Esophagus	2008267986	18.27	CBL	Missense_Mutation	p.C384S	26.90	NM_005188.3
49	1021	Esophagus	2008267986	18.27	BRCA2	Missense_Mutation	p.D1898G	45.60	NM_000059.3
49	1021	Esophagus	2008267986	18.27	SULT1A1	Missense_Mutation	p.F247L	9.00	NM_177530.2
49	1021	Esophagus	2008267986	18.27	CTCF	Missense_Mutation	p.M103I	2.90	NM_006565.3
49	1021	Esophagus	2008267986	18.27	ZFHX3	Missense_Mutation	p.A901S	40.20	NM_006885.3
49	1021	Esophagus	2008267986	18.27	PRKAR1A	Missense_Mutation	p.R211G	2.60	NM_212471.1
49	1021	Esophagus	2008267986	18.27	CRKL	Missense_Mutation	p.A208T	2.80	NM_005207.3
50	1021	Esophagus	2103106005	0.96	TP53	Frame_Shift	p.R280fs	68.73	NM_000546.5
50	1021	Esophagus	2103106005	0.96	DAXX	CN_Amp	NA	4.00	NM_001141970.1
52	295	Esophagus	AG1801130FFP	2.04	TP53	Missense_Mutation	p.I251N	20.62	NM_000546.5
52	295	Esophagus	AG1801130FFP	2.04	TP53	Missense_Mutation	p.S241Y	16.52	NM_000546.5
52	295	Esophagus	AG1801130FFP	2.04	SMARCB1	CN_Amp	NA	3.98	NM_003073.4
53	1021	Esophagus	2107151764	4.81	RB1	Splice_Site	c.1049+1G>T	53.10	NM_000321.2
53	1021	Esophagus	2107151764	4.81	PTPN11	Missense_Mutation	p.R498W	33.10	NM_002834.3
53	1021	Esophagus	2107151764	4.81	NF1	Frame_Shift	p.I679Dfs*21	23.60	NM_000267.3
53	1021	Esophagus	2107151764	4.81	KRAS	Missense_Mutation	p.G12V	20.50	NM_033360.2
53	1021	Esophagus	2107151764	4.81	PIK3CA	Missense_Mutation	p.H1047L	1.30	NM_006218.2
53	1021	Esophagus	2107151764	4.81	MYC	CN_Amp	NA	5.20	NM_002467.4
53	1021	Esophagus	2107151764	4.81	CDKN1A	CN_Amp	NA	4.60	NM_078467.2

53	1021	Esophagus	2107151764	4.81	DAXX	CN_Amp	NA	4.40	NM_001141970.1
53	1021	Esophagus	2107151764	4.81	EXT1	CN_Amp	NA	4.40	NM_000127.2
53	1021	Esophagus	2107151764	4.81	NBN	CN_Amp	NA	4.40	NM_002485.4
53	1021	Esophagus	2107151764	4.81	CCND3	CN_Amp	NA	4.20	NM_001760.3
53	1021	Esophagus	2107151764	4.81	DDR1	CN_Amp	NA	4.00	NM_001954.4
53	1021	Esophagus	2107151764	4.81	VEGFA	CN_Amp	NA	4.00	NM_001171623.1
53	1021	Esophagus	2107151764	4.81	RECQL4	CN_Amp	NA	4.00	NM_004260.3
54	295	Esophagus	AG1900071FFP	2.04	KRAS	Missense_Mutation	p.G12A	72.85	NM_033360.3
54	295	Esophagus	AG1900071FFP	2.04	FAT3	Frame_Shift	p.T1754fs	23.14	NM_001008781.2
54	295	Esophagus	AG1900071FFP	2.04	CHEK2	CN_Amp	NA	5.94	NM_007194.3
54	295	Esophagus	AG1900071FFP	2.04	EWSR1	CN_Amp	NA	5.14	NM_013986.3
54	295	Esophagus	AG1900071FFP	2.04	NF2	CN_Amp	NA	4.29	NM_000268.3
54	295	Esophagus	AG1900071FFP	2.04	EP300	CN_Amp	NA	4.09	NM_001429.3
55	1021	Esophagus	2103106004	2.88	AKT3	Missense_Mutation	p.L51R	5.40	NM_005465.4
55	1021	Esophagus	2103106004	2.88	AKT3	CN_Amp	NA	4.60	NM_005465.4
55	1021	Esophagus	2103106004	2.88	BRAF	Missense_Mutation	p.V600E	41.80	NM_004333.4
55	1021	Esophagus	2103106004	2.88	DDR2	CN_Amp	NA	4.40	NM_006182.2
55	1021	Esophagus	2103106004	2.88	FAT1	Missense_Mutation	p.V736L	55.70	NM_005245.3
55	1021	Esophagus	2103106004	2.88	IKBKE	CN_Amp	NA	4.40	NM_014002.3
55	1021	Esophagus	2103106004	2.88	NTRK1	CN_Amp	NA	4.80	NM_002529.3
55	1021	Esophagus	2103106004	2.88	PARP1	CN_Amp	NA	4.60	NM_001618.3
56	295	Esophagus	AG1801073FFP	6.12	NRAS	Missense_Mutation	p.G12V	58.26	NM_002524.4
56	295	Esophagus	AG1801073FFP	6.12	ERBB2	Missense_Mutation	p.R814C	43.34	NM_004448.3
56	295	Esophagus	AG1801073FFP	6.12	NOTCH2	CN_Amp	NA	3.78	NM_024408.3
56	295	Esophagus	AG1801073FFP	6.12	NRAS	CN_Amp	NA	9.52	NM_002524.4
56	295	Esophagus	AG1801073FFP	6.12	LRP1B	Missense_Mutation	p.R938H	59.08	NM_018557.2
56	295	Esophagus	AG1801073FFP	6.12	BARD1	Missense_Mutation	p.S131N	38.57	NM_000465.3
56	295	Esophagus	AG1801073FFP	6.12	MSH6	Missense_Mutation	p.I872T	76.02	NM_000179.2
56	295	Esophagus	AG1801073FFP	6.12	FANCL	Missense_Mutation	p.I354N	24.40	NM_018062.3
56	295	Esophagus	AG1801073FFP	6.12	FANCE	CN_Amp	NA	3.53	NM_021922.2
56	295	Esophagus	AG1801073FFP	6.12	IRF4	CN_Amp	NA	3.99	NM_002460.3
57	295	Esophagus	AG1900081FFP	3.06	STK11	Missense_Mutation	p.K78R	33.22	NM_000455.4
57	295	Esophagus	AG1900081FFP	3.06	NOTCH2	Missense_Mutation	p.A1654T	20.17	NM_024408.3
57	295	Esophagus	AG1900081FFP	3.06	NOTCH2	CN_Amp	NA	4.01	NM_024408.3
58	1021	Esophagus	2103186388	0.96	AURKA	CN_Amp	NA	4.60	NM_003600.2
58	1021	Esophagus	2103186388	0.96	BRD4	CN_Amp	NA	5.80	NM_058243.2
58	1021	Esophagus	2103186388	0.96	CCND3	CN_Amp	NA	5.00	NM_001760.3
58	1021	Esophagus	2103186388	0.96	CDC73	CN_Amp	NA	4.00	NM_024529.4
58	1021	Esophagus	2103186388	0.96	CIC	CN_Del	NA	0.20	NM_015125.3

58	1021	Esophagus	2103186388	0.96	DOT1L	CN_Amp	NA	5.00	NM_032482.2
58	1021	Esophagus	2103186388	0.96	GNA11	CN_Amp	NA	4.80	NM_002067.2
58	1021	Esophagus	2103186388	0.96	GNAS	CN_Amp	NA	4.60	NM_000516.4
58	1021	Esophagus	2103186388	0.96	HGF	Missense_Mutation	p.R134C	38.20	NM_000601.4
58	1021	Esophagus	2103186388	0.96	JAK3	CN_Amp	NA	4.60	NM_000215.3
58	1021	Esophagus	2103186388	0.96	KEAP1	CN_Amp	NA	4.40	NM_203500.1
58	1021	Esophagus	2103186388	0.96	MAP2K2	CN_Amp	NA	5.00	NM_030662.3
58	1021	Esophagus	2103186388	0.96	MDM4	CN_Amp	NA	4.20	NM_002393.4
58	1021	Esophagus	2103186388	0.96	MEF2B	CN_Amp	NA	4.80	NM_001145785.1
58	1021	Esophagus	2103186388	0.96	NOTCH3	CN_Amp	NA	4.40	NM_000435.2
58	1021	Esophagus	2103186388	0.96	PIK3R2	CN_Amp	NA	4.80	NM_005027.2
58	1021	Esophagus	2103186388	0.96	SMARCA4	CN_Amp	NA	4.80	NM_003072.3
58	1021	Esophagus	2103186388	0.96	SRC	CN_Amp	NA	4.20	NM_198291.1
58	1021	Esophagus	2103186388	0.96	STK11	CN_Amp	NA	4.00	NM_000455.4
58	1021	Esophagus	2103186388	0.96	TOP1	CN_Amp	NA	4.20	NM_003286.2
58	1021	Esophagus	2103186388	0.96	VEGFA	CN_Amp	NA	5.20	NM_001171623.1
59	295	Esophagus	AG1801126FFP	5.10	RB1	Missense_Mutation	p.G449E	24.77	NM_000321.2
59	295	Esophagus	AG1801126FFP	5.10	RB1	Nonsense_Mutation	p.R552*	59.65	NM_000321.2
59	295	Esophagus	AG1801126FFP	5.10	KMT2D	Frame_Shift	p.P2948fs	31.34	NM_003482.3
59	295	Esophagus	AG1801126FFP	5.10	SMO	Missense_Mutation	p.D782Y	36.88	NM_005631.4
59	295	Esophagus	AG1801126FFP	5.10	NBN	CN_Amp	NA	4.81	NM_002485.4
59	295	Esophagus	AG1801126FFP	5.10	NOTCH1	CN_Amp	NA	3.58	NM_017617.4
59	295	Esophagus	AG1801126FFP	5.10	BCORL1	Frame_Shift	p.P214fs	64.04	NM_021946.4
60	295	Esophagus	AG1801074FFP	2.04	TP53	Splice_Site	p.S367=	10.84	NM_000546.5
60	295	Esophagus	AG1801074FFP	2.04	NOTCH1	Frame_Shift	p.C1414fs	5.31	NM_017617.4
61	1021	Esophagus	2103186387	1.92	KDR	CN_Amp	NA	11.00	NM_002253.2
61	1021	Esophagus	2103186387	1.92	KIT	Missense_Mutation	p.L576P	93.60	NM_000222.2
61	1021	Esophagus	2103186387	1.92	KIT	CN_Amp	NA	12.40	NM_000222.2
61	1021	Esophagus	2103186387	1.92	PDGFRA	CN_Amp	NA	NA	NM_006206.4
61	1021	Esophagus	2103186387	1.92	PMS2	CN_Amp	NA	4.40	NM_000535.5
61	1021	Esophagus	2103186387	1.92	SF3B1	Missense_Mutation	p.R625C	25.00	NM_012433.2
62	295	Esophagus	AG1900909FFP	3.06	NRAS	Missense_Mutation	p.G13D	35.50	NM_002524.4
62	295	Esophagus	AG1900909FFP	3.06	RAD51C	In_Frame	p.K186dup	31.20	NM_058216.2
62	295	Esophagus	AG1900909FFP	3.06	DNMT3A	Splice_Site	c.2322+3A>G	5.88	NM_022552.4
62	295	Esophagus	AG1900909FFP	3.06	ASXL1	CN_Amp	NA	4.12	NM_015338.5
63	1021	Esophagus	2010220398	4.81	KIT	Missense_Mutation	p.V559A	88.60	NM_000222.2
63	1021	Esophagus	2010220398	4.81	SF3B1	Missense_Mutation	p.R625H	30.40	NM_012433.2
63	1021	Esophagus	2010220398	4.81	RHOA	Missense_Mutation	p.G166R	30.50	NM_001664.2
63	1021	Esophagus	2010220398	4.81	ARID2	Frame_Shift	p.A1807Lfs*32	29.70	NM_152641.2

63	1021	Esophagus	2010220398	4.81	ZFHX3	Missense_Mutation	p.Q1737K	15.00	NM_006885.3
63	1021	Esophagus	2010220398	4.81	KIT	CN_Amp	NA	11.26	NM_000222.2
64	1021	Small bowel	2009239326	2.88	NOTCH1	Missense_Mutation	p.E1450K	37.80	NM_017617.3
64	1021	Small bowel	2009239326	2.88	PIK3C2G	Missense_Mutation	p.D1389N	23.40	NM_004570.4
64	1021	Small bowel	2009239326	2.88	NSD1	Missense_Mutation	p.L889F	13.50	NM_022455.4
65	1021	Small bowel	2010230472	13.46	BRAF	In_Frame	p.T599_V600insT	26.20	NM_004333.4
65	1021	Small bowel	2010230472	13.46	BRAF	In_Frame	p.T244_L245delinsS	23.70	NM_004333.4
65	1021	Small bowel	2010230472	13.46	BCOR	Missense_Mutation	p.T877I	76.20	NM_001123385.1
65	1021	Small bowel	2010230472	13.46	OR6F1	Missense_Mutation	p.L258F	72.80	NM_001005286.1
65	1021	Small bowel	2010230472	13.46	PTPRD	Missense_Mutation	p.E678K	42.20	NM_002839.3
65	1021	Small bowel	2010230472	13.46	GKN2	Missense_Mutation	p.E152K	32.50	NM_182536.2
65	1021	Small bowel	2010230472	13.46	RHOA	Frame_Shift	p.L179Rfs*4	32.50	NM_001664.2
65	1021	Small bowel	2010230472	13.46	RHOA	Missense_Mutation	p.L22H	26.90	NM_001664.2
65	1021	Small bowel	2010230472	13.46	LRP1B	Missense_Mutation	p.I4378T	31.04	NM_018557.2
65	1021	Small bowel	2010230472	13.46	NCOR1	Missense_Mutation	p.T952I	31.20	NM_006311.3
65	1021	Small bowel	2010230472	13.46	IL7R	Missense_Mutation	p.S366F	28.90	NM_002185.3
65	1021	Small bowel	2010230472	13.46	ROS1	Missense_Mutation	p.S418L	27.60	NM_002944.2
65	1021	Small bowel	2010230472	13.46	ERBB4	Missense_Mutation	p.E452K	27.00	NM_005235.2
65	1021	Small bowel	2010230472	13.46	TERT	5'UTR	c.-58-u68_-58-u66delCCCinsTCT	24.80	NM_198253.2
66	1021	Anorectum	2010160113	2.88	MTOR	Nonsense_Mutation	p.R2443*	1.10	NM_004958.3
66	1021	Anorectum	2010160113	2.88	EPHB1	Missense_Mutation	p.R868Q	1.20	NM_004441.4
66	1021	Anorectum	2010160113	2.88	ATM	In_Frame	p.D2448_L2452delinsV	24.20	NM_000051.3
66	1021	Anorectum	2010160113	2.88	MYC	CN_Amp	NA	4.35	NM_002467.4
67	1021	Anorectum	2010230471	1.92	TP53	Missense_Mutation	p.R175H	14.10	NM_000546.5
67	1021	Anorectum	2010230471	1.92	ATRX	Frame_Shift	p.L563Yfs*11	11.40	NM_000489.3
67	1021	Anorectum	2010230471	1.92	BRCA2	Frame_Shift	p.W1692Mfs*3	NA	NM_000059.3
68	1021	Anorectum	1908153669	3.85	TERT	5'UTR	c.-124C>T	35.70	NM_198253.2
68	1021	Anorectum	1908153669	3.85	FLG	Missense_Mutation	p.R2857H	10.30	NM_002016.1
68	1021	Anorectum	1908153669	3.85	FMN2	Missense_Mutation	p.G950R	15.70	NM_020066.4
68	1021	Anorectum	1908153669	3.85	PIK3R1	Frame_Shift	p.G665Afs*3	17.10	NM_181523.2
68	1021	Anorectum	1908153669	3.85	TPM3:ASXL2	Fusion	NA	4.10	NM_001043353.1; NM_015338.5
68	1021	Anorectum	1908153669	3.85	IFNG	CN_Del	NA	0.68	NM_000619.2
68	1021	Anorectum	1908153669	3.85	RAD51	CN_Del	NA	0.67	NM_002875.4
69	1021	Anorectum	1910256447	10.58	NCF2	Missense_Mutation	p.E453K	17.30	NM_000433.3
69	1021	Anorectum	1910256447	10.58	DNMT3A	Frame_Shift	p.Q29Hfs*36	16.70	NM_022552.4
69	1021	Anorectum	1910256447	10.58	FGFR4	Missense_Mutation	p.I281S	10.60	NM_213647.1
69	1021	Anorectum	1910256447	10.58	ARID1B	Missense_Mutation	p.N1463S	11.70	NM_020732.3
69	1021	Anorectum	1910256447	10.58	GPR124	Frame_Shift	p.F654Sfs*51	19.40	NM_032777.9

69	1021	Anorectum	1910256447	10.58	GPR124	Missense_Mutation	p.S656R	19.80	NM_032777.9
69	1021	Anorectum	1910256447	10.58	FANCG	Missense_Mutation	p.V29L	10.80	NM_004629.1
69	1021	Anorectum	1910256447	10.58	ATM	Missense_Mutation	p.L1617R	22.20	NM_000051.3
69	1021	Anorectum	1910256447	10.58	MLL2	Missense_Mutation	p.E219K	11.04	NM_003482.3
69	1021	Anorectum	1910256447	10.58	SOX9	Missense_Mutation	p.E13G	8.80	NM_000346.3
69	1021	Anorectum	1910256447	10.58	CHEK2	Missense_Mutation	p.K373E	17.30	NM_007194.3
69	1021	Anorectum	1910256447	10.58	MYC	CN_Amp	NA	7.68	NM_002467.4
70	1021	Anorectum	2006094452	0.96	HIST1H3I	Missense_Mutation	p.R132L	14.50	NM_003533.2
70	1021	Anorectum	2006094452	0.96	KRAS	CN_Amp	NA	43.90	NM_033360.2
70	1021	Anorectum	2006094452	0.96	CDK4	CN_Amp	NA	8.58	NM_000075.3
70	1021	Anorectum	2006094452	0.96	MDM2	CN_Amp	NA	5.07	NM_002392.4
70	1021	Anorectum	2006094452	0.96	RB1	CN_Amp	NA	4.86	NM_000321.2
71	1021	Anorectum	2010160112	1.92	BRAF	Missense_Mutation	p.D594N	1.04	NM_004333.4
71	1021	Anorectum	2010160112	1.92	NF1	Splice_Site	c.1063-1G>T	56.00	NM_000267.3
71	1021	Anorectum	2010160112	1.92	PMS2	CN_Amp	NA	3.85	NM_000535.5
71	1021	Anorectum	2010160112	1.92	MET	CN_Amp	NA	3.57	NM_000245.2
71	1021	Anorectum	2010160112	1.92	BRAF	CN_Amp	NA	3.87	NM_004333.4
72	295	Anorectum	AG1800805FFP	1.02	GNA13	Missense_Mutation	p.K204N	5.13	NM_006572.5
72	295	Anorectum	AG1800805FFP	1.02	NFE2L2	CN_Amp	NA	4.00	NM_006164.4
72	295	Anorectum	AG1800805FFP	1.02	STAT4	CN_Amp	NA	3.79	NM_001243835.1
72	295	Anorectum	AG1800805FFP	1.02	SF3B1	CN_Amp	NA	4.04	NM_001005526.2
72	295	Anorectum	AG1800805FFP	1.02	RPTOR	CN_Amp	NA	4.21	NM_020761.2
73	295	Anorectum	AG1800183FFP	6.12	KIT	Missense_Mutation	p.L576P	56.51	NM_000222.2
73	295	Anorectum	AG1800183FFP	6.12	NOTCH3	Missense_Mutation	p.R1857W	20.39	NM_000435.2
73	295	Anorectum	AG1800183FFP	6.12	ROS1	Missense_Mutation	p.L489I	38.48	NM_002944.2
73	295	Anorectum	AG1800183FFP	6.12	JAK2	Missense_Mutation	p.E338G	12.50	NM_004972.3
73	295	Anorectum	AG1800183FFP	6.12	LRP1B	Missense_Mutation	p.A545V	9.91	NM_018557.2
73	295	Anorectum	AG1800183FFP	6.12	ABL1	Frame_Shift	p.Q252fs	9.52	NM_005157.5
73	295	Anorectum	AG1800183FFP	6.12	PDGFRA	CN_Amp	NA	4.13	NM_006206.4
73	295	Anorectum	AG1800183FFP	6.12	KIT	CN_Amp	NA	5.41	NM_000222.2
73	295	Anorectum	AG1800183FFP	6.12	KDR	CN_Amp	NA	4.81	NM_002253.2
73	295	Anorectum	AG1800183FFP	6.12	ERBB2	CN_Del	NA	1.10	NM_004448.3
74	295	Anorectum	AG1800172FFP	5.10	NRAS	Missense_Mutation	p.G12D	4.88	NM_002524.4
74	295	Anorectum	AG1800172FFP	5.10	PTEN	Missense_Mutation	p.I50N	10.87	NM_000314.6
74	295	Anorectum	AG1800172FFP	5.10	BRCA2	Missense_Mutation	p.Q2345E	9.89	NM_000059.3
74	295	Anorectum	AG1800172FFP	5.10	NCOR1	Missense_Mutation	p.P1994S	15.87	NM_006311.3
74	295	Anorectum	AG1800172FFP	5.10	GNA11	Missense_Mutation	p.R183L	11.25	NM_002067.4
75	295	Anorectum	AG1800605FFP	2.04	ATM	Missense_Mutation	p.S2017R	36.16	NM_000051.3
75	295	Anorectum	AG1800605FFP	2.04	FGFR2	Missense_Mutation	p.S299N	4.58	NM_000141.4

75	295	Anorectum	AG1800605FFP	2.04	PIK3C2G	CN_Amp	NA	8.34	NM_001288772.1
75	295	Anorectum	AG1800605FFP	2.04	KIT	CN_Amp	NA	5.38	NM_000222.2
75	295	Anorectum	AG1800605FFP	2.04	KDR	CN_Amp	NA	4.94	NM_002253.2
76	295	Anorectum	AG1900875FFP	5.10	KRAS	Missense_Mutation	p.G12A	7.85	NM_033360.3
76	295	Anorectum	AG1900875FFP	5.10	ERBB3	Missense_Mutation	p.E928G	2.02	NM_001982.3
76	295	Anorectum	AG1900875FFP	5.10	PARP4	Missense_Mutation	p.L1162V	5.87	NM_006437.3
76	295	Anorectum	AG1900875FFP	5.10	CREBBP	Missense_Mutation	p.D1273E	4.91	NM_004380.2
76	295	Anorectum	AG1900875FFP	5.10	TERT	5'UTR	c.124C>T	NA	NM_198253.2
77	1021	Anorectum	2008137407	8.65	BCOR	Missense_Mutation	p.P405L	65.70	NM_001123385.1
77	1021	Anorectum	2008137407	8.65	BARD1	Missense_Mutation	p.K754E	52.70	NM_000465.2
77	1021	Anorectum	2008137407	8.65	ASPM	In_Frame	p.N508_Q509insP	51.90	NM_018136.4
77	1021	Anorectum	2008137407	8.65	CDK13	Missense_Mutation	p.P83S	45.00	NM_003718.4
77	1021	Anorectum	2008137407	8.65	GRIN2A	Missense_Mutation	p.I763V	44.70	NM_001134407.1
77	1021	Anorectum	2008137407	8.65	SPSB4	Missense_Mutation	p.A172V	44.50	NM_080862.1
77	1021	Anorectum	2008137407	8.65	PMS1	Missense_Mutation	p.R919C	42.80	NM_000534.4
77	1021	Anorectum	2008137407	8.65	MEN1	Frame_Shift	p.F93Lfs*24	42.30	NM_130799.2
77	1021	Anorectum	2008137407	8.65	TRIM58	Missense_Mutation	p.L232Q	39.80	NM_015431.3
77	1021	Anorectum	2008137407	8.65	CCND1	CN_Amp	NA	6.79	NM_053056.2
77	1021	Anorectum	2008137407	8.65	FGF19	CN_Amp	NA	5.89	NM_005117.2
77	1021	Anorectum	2008137407	8.65	FGF4	CN_Amp	NA	6.87	NM_002007.2
77	1021	Anorectum	2008137407	8.65	FGF3	CN_Amp	NA	6.30	NM_005247.2
77	1021	Anorectum	2008137407	8.65	C11orf30	CN_Amp	NA	6.35	NM_020193.3
77	1021	Anorectum	2008137407	8.65	BRCA2	CN_Amp	NA	4.89	NM_000059.3
77	1021	Anorectum	2008137407	8.65	KATNAL1-N4BP2L1	Fusion	NA	4.50	NM_032116.5;NM_052818.2
78	1021	Anorectum	1910186181	9.62	NF1	Frame_Shift	p.S1355Ffs*19	54.60	NM_000267.3
78	1021	Anorectum	1910186181	9.62	RHOA	Missense_Mutation	p.K186E	19.30	NM_001664.2
78	1021	Anorectum	1910186181	9.62	IL7R	Missense_Mutation	p.L370M	4.40	NM_002185.3
78	1021	Anorectum	1910186181	9.62	PTEN	Missense_Mutation	p.C71F	1.80	NM_000314.4
78	1021	Anorectum	1910186181	9.62	PTEN	Frame_Shift	p.N262Qfs*35	5.50	NM_000314.4
78	1021	Anorectum	1910186181	9.62	MLL2	Missense_Mutation	p.P1162L	59.80	NM_003482.3
78	1021	Anorectum	1910186181	9.62	BRCA2	Missense_Mutation	p.Y3009C	4.10	NM_000059.3
78	1021	Anorectum	1910186181	9.62	NTRK3	Missense_Mutation	p.F395S	2.50	NM_001012338.2
78	1021	Anorectum	1910186181	9.62	CREBBP	Missense_Mutation	p.G2401E	19.40	NM_004380.2
78	1021	Anorectum	1910186181	9.62	CYLD	Missense_Mutation	p.R394C	1.80	NM_001042355.1
79	295	Anorectum	AG1700430FFP	13.27	CBL	Missense_Mutation	p.C396F	45.40	NM_005188.3
79	295	Anorectum	AG1700430FFP	13.27	CDK12	Missense_Mutation	p.S1236N	75.80	NM_016507.3
79	295	Anorectum	AG1700430FFP	13.27	HLA-A	Missense_Mutation	p.F33T	25.70	NM_001242758.1
79	295	Anorectum	AG1700430FFP	13.27	ROS1	Splice_Site	NA	67.20	NM_002944.2

79	295	Anorectum	AG1700430FFP	13.27	SMO	Missense_Mutation	p.T336I	25.10	NM_005631.4
79	295	Anorectum	AG1700430FFP	13.27	PRKDC	Missense_Mutation	p.Q3633E	46.50	NM_006904.6
79	295	Anorectum	AG1700430FFP	13.27	ATRX	Missense_Mutation	p.L1592V	26.10	NM_000489.4
79	295	Anorectum	AG1700430FFP	13.27	GNAS	Missense_Mutation	p.R874H	2.20	NM_080425.3
79	295	Anorectum	AG1700430FFP	13.27	NF2	Missense_Mutation	p.G302R	4.40	NM_000268.3
79	295	Anorectum	AG1700430FFP	13.27	NUP93	Splice_Site	NA	2.40	NM_014669.4
79	295	Anorectum	AG1700430FFP	13.27	CTCF	Frame_Shift	p.T317fs	2.30	NM_006565.3
79	295	Anorectum	AG1700430FFP	13.27	NBN	Missense_Mutation	p.A131V	2.40	NM_002485.4
79	295	Anorectum	AG1700430FFP	13.27	NBN	In_Frame	p.N414del	3.10	NM_002485.4
80	1021	Anorectum	1909235233	7.69	ATRX	Nonsense_Mutation	p.L1341*	30.40	NM_000489.3
80	1021	Anorectum	1909235233	7.69	KIT	In_Frame	p.Y503_F504insAY	19.50	NM_000222.2
80	1021	Anorectum	1909235233	7.69	HOXB13	Missense_Mutation	p.R267C	13.70	NM_006361.5
80	1021	Anorectum	1909235233	7.69	SUCLG1	Missense_Mutation	p.I143N	10.80	NM_003849.3
80	1021	Anorectum	1909235233	7.69	DPYD	Missense_Mutation	p.L741V	10.00	NM_000110.3
80	1021	Anorectum	1909235233	7.69	ZFHX3	Missense_Mutation	p.Q3197R	7.30	NM_006885.3
80	1021	Anorectum	1909235233	7.69	FAT2	Missense_Mutation	p.R543C	4.30	NM_001447.2
80	1021	Anorectum	1909235233	7.69	MSH3	Missense_Mutation	p.A58P	5.90	NM_002439.4
81	1021	Anorectum	2010220418	11.54	TET2	Missense_Mutation	p.R814C	54.70	NM_001127208.2
81	1021	Anorectum	2010220418	11.54	BRCA1	Missense_Mutation	p.L1414V	52.90	NM_007294.3
81	1021	Anorectum	2010220418	11.54	ARAF	Frame_Shift	p.P595Vfs*44	51.30	NM_001654.4
81	1021	Anorectum	2010220418	11.54	TBX3	Missense_Mutation	p.E122D	49.90	NM_016569.3
81	1021	Anorectum	2010220418	11.54	FAT1	Missense_Mutation	p.P1509L	43.60	NM_005245.3
81	1021	Anorectum	2010220418	11.54	CDKN2A	Splice_Site	c.457+1G>C	27.70	NM_000077.4
81	1021	Anorectum	2010220418	11.54	OR5L2	Missense_Mutation	p.T46I	26.60	NM_001004739.1
81	1021	Anorectum	2010220418	11.54	BRIP1	Missense_Mutation	p.T113I	19.20	NM_032043.2
81	1021	Anorectum	2010220418	11.54	FLT1	Missense_Mutation	p.F362I	18.80	NM_002019.4
81	1021	Anorectum	2010220418	11.54	HIST1H2BK	Missense_Mutation	p.A22V	18.10	NM_080593.2
81	1021	Anorectum	2010220418	11.54	ROS1	Missense_Mutation	p.Q237E	10.30	NM_002944.2
81	1021	Anorectum	2010220418	11.54	EPHB6	Missense_Mutation	p.R777W	7.70	NM_004445.3
81	1021	Anorectum	2010220418	11.54	PTPN11	CN_Amp	NA	5.00	NM_002834.3
81	1021	Anorectum	2010220418	11.54	SDHA	CN_Amp	NA	4.20	NM_004168.2
81	1021	Anorectum	2010220418	11.54	CARD11-SDK1	Fusion	NA	6.00	NM_032415.4;NM_152744.4
82	295	Genitourinary tract	AG1900654FFP	3.06	APC	Frame_Shift	p.L589fs	59.82	NM_000038.5
82	295	Genitourinary tract	AG1900654FFP	3.06	GRIN2A	Missense_Mutation	p.M133V	26.32	NM_000833.4
82	295	Genitourinary tract	AG1900654FFP	3.06	PRKDC	Missense_Mutation	p.T874S	20.80	NM_006904.6
83	1021	Genitourinary tract	2006164751	2.88	TERT	5'UTR	c.124C>T	37.80	NM_198253.2
83	1021	Genitourinary tract	2006164751	2.88	BRAF	Missense_Mutation	p.E501G	25.00	NM_004333.4
83	1021	Genitourinary tract	2006164751	2.88	GNA11	Missense_Mutation	p.I62S	14.00	NM_002067.2

85	1021	Genitourinary tract	2008177548	3.85	RET	Missense_Mutation	p.S131T	31.90	NM_020975.4
85	1021	Genitourinary tract	2008177548	3.85	FAT2	Missense_Mutation	p.L1105W	29.30	NM_001447.2
85	1021	Genitourinary tract	2008177548	3.85	EXT1	Missense_Mutation	p.R440H	23.40	NM_000127.2
85	1021	Genitourinary tract	2008177548	3.85	DDR2	Missense_Mutation	p.R31S	22.30	NM_006182.2
85	1021	Genitourinary tract	2008177548	3.85	NBN	CN_Amp	NA	4.00	NM_002485.4
85	1021	Genitourinary tract	2008177548	3.85	MYC	CN_Amp	NA	5.80	NM_002467.4
85	1021	Genitourinary tract	2008177548	3.85	RECQL4	CN_Amp	NA	4.20	NM_004260.3
85	1021	Genitourinary tract	2008177548	3.85	ERBB2	CN_Amp	NA	16.80	NM_004448.2
87	1021	Genitourinary tract	2006235098	4.81	NRAS	Missense_Mutation	p.G13D	1.50	NM_002524.4
87	1021	Genitourinary tract	2006235098	4.81	TERT	5'UTR	c.124C>T	28.50	NM_198253.2
87	1021	Genitourinary tract	2006235098	4.81	PTEN	Frame_Shift	p.L316Ffs*5	31.04	NM_000314.4
87	1021	Genitourinary tract	2006235098	4.81	PTPN11	Missense_Mutation	p.A307V	14.80	NM_002834.3
87	1021	Genitourinary tract	2006235098	4.81	GRIN2A	Missense_Mutation	p.K1281T	5.30	NM_001134407.1
87	1021	Genitourinary tract	2006235098	4.81	CDK4	CN_Amp	NA	7.27	NM_000075.3
87	1021	Genitourinary tract	2006235098	4.81	MDM2	CN_Amp	NA	6.33	NM_002392.4
87	1021	Genitourinary tract	2006235098	4.81	IFNG	CN_Amp	NA	5.24	NM_000619.2
88	1021	Genitourinary tract	2002040439	2.88	TP53	Missense_Mutation	p.Y163C	71.04	NM_000546.5
88	1021	Genitourinary tract	2002040439	2.88	EPHB2	Missense_Mutation	p.G282E	11.04	NM_017449.3
88	1021	Genitourinary tract	2002040439	2.88	FAT1	Missense_Mutation	p.D2158V	10.50	NM_005245.3
89	1021	Genitourinary tract	2001220357	4.81	JAK2	Nonsense_Mutation	p.Q419*	62.90	NM_004972.3
89	1021	Genitourinary tract	2001220357	4.81	NF2	Missense_Mutation	p.A370P	34.00	NM_000268.3
89	1021	Genitourinary tract	2001220357	4.81	JAK3	Missense_Mutation	p.T75M	33.20	NM_000215.3
89	1021	Genitourinary tract	2001220357	4.81	SLIT2	Missense_Mutation	p.K1322R	17.10	NM_004787.1
89	1021	Genitourinary tract	2001220357	4.81	CDH1	Missense_Mutation	p.L416F	1.90	NM_004360.3
89	1021	Genitourinary tract	2001220357	4.81	PIK3CB	CN_Amp	NA	8.12	NM_006219.2
89	1021	Genitourinary tract	2001220357	4.81	ATR	CN_Amp	NA	8.16	NM_001184.3
90	295	Genitourinary tract	AG1800379FFP	7.14	KIT	Missense_Mutation	p.W557G	58.21	NM_000222.2
90	295	Genitourinary tract	AG1800379FFP	7.14	KDR	Missense_Mutation	p.C1007S	60.53	NM_002253.2
90	295	Genitourinary tract	AG1800379FFP	7.14	LRP1B	Missense_Mutation	p.D653N	36.61	NM_018557.2
90	295	Genitourinary tract	AG1800379FFP	7.14	ALK	Missense_Mutation	p.R311H	53.27	NM_004304.4
90	295	Genitourinary tract	AG1800379FFP	7.14	KEAP1	Missense_Mutation	p.A454V	15.12	NM_012289.3
90	295	Genitourinary tract	AG1800379FFP	7.14	FANCC	Missense_Mutation	p.G148R	40.16	NM_000136.2
90	295	Genitourinary tract	AG1800379FFP	7.14	STAG2	Missense_Mutation	p.D158H	37.11	NM_001042749.2
90	295	Genitourinary tract	AG1800379FFP	7.14	SLC16A7-MDM2	Fusion	NA	2.00	NM_001270623.2; NM_002392.5
90	295	Genitourinary tract	AG1800379FFP	7.14	ERBB3	CN_Amp	NA	3.77	NM_001982.3
90	295	Genitourinary tract	AG1800379FFP	7.14	CDK4	CN_Amp	NA	16.70	NM_000075.3
90	295	Genitourinary tract	AG1800379FFP	7.14	MDM2	CN_Amp	NA	11.31	NM_002392.5
90	295	Genitourinary tract	AG1800379FFP	7.14	GNA13	CN_Amp	NA	4.16	NM_006572.5

90	295	Genitourinary tract	AG1800379FFP	7.14	PRKAR1A	CN_Amp	NA	3.97	NM_002734.4
90	295	Genitourinary tract	AG1800379FFP	7.14	CCNE1	CN_Amp	NA	5.59	NM_001238.3
90	295	Genitourinary tract	AG1800379FFP	7.14	SPEN	CN_Amp	NA	3.69	NM_015001.2
90	295	Genitourinary tract	AG1800379FFP	7.14	MTOR	CN_Amp	NA	4.35	NM_004958.3
90	295	Genitourinary tract	AG1800379FFP	7.14	TNFRSF14	CN_Amp	NA	3.66	NM_003820.3
90	295	Genitourinary tract	AG1800379FFP	7.14	TOP1	CN_Amp	NA	3.85	NM_003286.2
90	295	Genitourinary tract	AG1800379FFP	7.14	MITF	CN_Amp	NA	5.17	NM_000248.3
90	295	Genitourinary tract	AG1800379FFP	7.14	PDGFRA	CN_Amp	NA	6.43	NM_006206.4
90	295	Genitourinary tract	AG1800379FFP	7.14	KIT	CN_Amp	NA	6.70	NM_000222.2
90	295	Genitourinary tract	AG1800379FFP	7.14	KDR	CN_Amp	NA	5.16	NM_002253.2
90	295	Genitourinary tract	AG1800379FFP	7.14	RICTOR	CN_Amp	NA	3.68	NM_001285439.1
90	295	Genitourinary tract	AG1800379FFP	7.14	IRF4	CN_Amp	NA	4.75	NM_002460.3
90	295	Genitourinary tract	AG1800379FFP	7.14	EGFR	CN_Del	NA	0.93	NM_005228.3
90	295	Genitourinary tract	AG1800379FFP	7.14	HGF	CN_Del	NA	0.87	NM_000601.5
91	295	Genitourinary tract	AG1800038FFP	2.04	PTEN	Nonsense_Mutation	p.R84*	79.60	NM_000314.6
91	295	Genitourinary tract	AG1800038FFP	2.04	KAT6A	Missense_Mutation	p.E796A	3.30	NM_006766.4
91	295	Genitourinary tract	AG1800038FFP	2.04	KDR	CN_Amp	NA	7.34	NM_002253.2
91	295	Genitourinary tract	AG1800038FFP	2.04	KIT	CN_Amp	NA	7.40	NM_000222.2
91	295	Genitourinary tract	AG1800038FFP	2.04	PDGFRA	CN_Amp	NA	9.31	NM_006206.4
91	295	Genitourinary tract	AG1800038FFP	2.04	EPHA5	CN_Amp	NA	5.24	NM_001281765.2
91	295	Genitourinary tract	AG1800038FFP	2.04	KDM5A	CN_Amp	NA	7.53	NM_001042603.2
91	295	Genitourinary tract	AG1800038FFP	2.04	ERBB3	CN_Amp	NA	6.28	NM_001982.3
91	295	Genitourinary tract	AG1800038FFP	2.04	CDK4	CN_Amp	NA	20.66	NM_000075.3
91	295	Genitourinary tract	AG1800038FFP	2.04	MDM2	CN_Amp	NA	6.25	NM_002392.5
92	295	Genitourinary tract	AG1800265FFP	2.04	TP53	Missense_Mutation	p.S241Y	56.92	NM_000546.5
92	295	Genitourinary tract	AG1800265FFP	2.04	ATRX	Frame_Shift	p.K773fs	53.93	NM_000489.4
93	295	Genitourinary tract	AG1700457FFP	2.04	CARD11	In_Frame	p.S622del	2.80	NM_032415.5
93	295	Genitourinary tract	AG1700457FFP	2.04	PIK3C3	Missense_Mutation	p.D852G	9.30	NM_002647.3
93	295	Genitourinary tract	AG1700457FFP	2.04	KDR	CN_Amp	NA	3.57	NM_002253.2
94	295	Genitourinary tract	AG1700530FFP	5.10	TP53	Frame_Shift	p.P85fs	83.00	NM_000546.5
94	295	Genitourinary tract	AG1700530FFP	5.10	MLH1	Missense_Mutation	p.S95A	84.90	NM_000249.3
94	295	Genitourinary tract	AG1700530FFP	5.10	BRCA2	Missense_Mutation	p.N72S	9.00	NM_000059.3
94	295	Genitourinary tract	AG1700530FFP	5.10	PTEN	Missense_Mutation	p.A39V	4.20	NM_000314.6
94	295	Genitourinary tract	AG1700530FFP	5.10	TERT	5'UTR	c.124C>T	NA	NM_198253.2
94	295	Genitourinary tract	AG1700530FFP	5.10	NOTCH4	CN_Amp	NA	4.20	NM_004557.3
94	295	Genitourinary tract	AG1700530FFP	5.10	IGF1R	CN_Amp	NA	6.86	NM_002392.5
94	295	Genitourinary tract	AG1700530FFP	5.10	NCOR1	CN_Amp	NA	4.06	NM_006311.3
94	295	Genitourinary tract	AG1700530FFP	5.10	ALOX12B	CN_Del	NA	0.31	NM_001139.2
94	295	Genitourinary tract	AG1700530FFP	5.10	TOP1	CN_Amp	NA	3.54	NM_003286.2

95	295	Genitourinary tract	AG1700544FFP	2.04	PTEN	Splice_Site	NA	47.60	NM_000314.6
95	295	Genitourinary tract	AG1700544FFP	2.04	LRP1B	Missense_Mutation	p.I3410N	43.10	NM_018557.2
95	295	Genitourinary tract	AG1700544FFP	2.04	DAXX	CN_Amp	NA	6.18	NM_001141970.1
95	295	Genitourinary tract	AG1700544FFP	2.04	IRF4	CN_Amp	NA	4.71	NM_002460.3
95	295	Genitourinary tract	AG1700544FFP	2.04	PNRC1	CN_Amp	NA	4.93	NM_006813.2
95	295	Genitourinary tract	AG1700544FFP	2.04	ESR1	CN_Del	NA	1.01	NM_000125.3
95	295	Genitourinary tract	AG1700544FFP	2.04	CARD11	CN_Amp	NA	3.97	NM_032415.5
95	295	Genitourinary tract	AG1700544FFP	2.04	CDK6	CN_Amp	NA	9.00	NM_001145306.1
95	295	Genitourinary tract	AG1700544FFP	2.04	MET	CN_Amp	NA	5.61	NM_000245.3
95	295	Genitourinary tract	AG1700544FFP	2.04	FANCG	CN_Amp	NA	4.51	NM_004629.1
95	295	Genitourinary tract	AG1700544FFP	2.04	CHUK	CN_Amp	NA	5.14	NM_001278.4
95	295	Genitourinary tract	AG1700544FFP	2.04	FGF4	CN_Del	NA	0.78	NM_002007.2
95	295	Genitourinary tract	AG1700544FFP	2.04	CDK4	CN_Amp	NA	3.92	NM_000075.3
95	295	Genitourinary tract	AG1700544FFP	2.04	NTRK3	CN_Del	NA	0.89	NM_001012338.2
95	295	Genitourinary tract	AG1700544FFP	2.04	JAK3	CN_Amp	NA	3.57	NM_000215.3
95	295	Genitourinary tract	AG1700544FFP	2.04	PIK3R2	CN_Amp	NA	3.96	NM_005027.3
95	295	Genitourinary tract	AG1700544FFP	2.04	STK11	CN_Amp	NA	3.78	NM_000455.4
95	295	Genitourinary tract	AG1700544FFP	2.04	SOX10	CN_Amp	NA	4.52	NM_006941.3
96	295	Genitourinary tract	AG1700545FFP	8.16	FANCD2	Missense_Mutation	p.I517V	76.70	NM_001018115.2
96	295	Genitourinary tract	AG1700545FFP	8.16	CYP17A1	Nonsense_Mutation	p.W499*	77.80	NM_000102.3
96	295	Genitourinary tract	AG1700545FFP	8.16	DNMT3A	Missense_Mutation	p.D876N	43.80	NM_022552.4
96	295	Genitourinary tract	AG1700545FFP	8.16	ZNF703	Missense_Mutation	p.Y574C	24.00	NM_025069.2
96	295	Genitourinary tract	AG1700545FFP	8.16	TP53	Splice_Site	NA	17.50	NM_000546.5
96	295	Genitourinary tract	AG1700545FFP	8.16	FANCI	Missense_Mutation	p.K1290Q	10.90	NM_001113378.1
96	295	Genitourinary tract	AG1700545FFP	8.16	PRKDC	Missense_Mutation	p.R3045L	14.60	NM_006904.6
96	295	Genitourinary tract	AG1700545FFP	8.16	PIK3R1	In_Frame	p.W583del	8.30	NM_181523.2
96	295	Genitourinary tract	AG1700545FFP	8.16	EMSY	CN_Amp	NA	6.73	NM_001300942.1
96	295	Genitourinary tract	AG1700545FFP	8.16	DIS3	CN_Amp	NA	6.41	NM_014953.4
97	295	Genitourinary tract	AG1700539FFP	1.02	SETD2	Missense_Mutation	p.A1382S	52.10	NM_014159.6
98	1021	Genitourinary tract	1910105767	3.85	PIK3CB	Missense_Mutation	p.N553S	87.50	NM_006219.2
98	1021	Genitourinary tract	1910105767	3.85	ATR	Missense_Mutation	p.S1801N	85.80	NM_001184.3
98	1021	Genitourinary tract	1910105767	3.85	MCL1	Missense_Mutation	p.S162L	40.30	NM_021960.4
98	1021	Genitourinary tract	1910105767	3.85	FAT2	Missense_Mutation	p.V2307I	3.90	NM_001447.2
98	1021	Genitourinary tract	1910105767	3.85	SDHA	CN_Amp	NA	7.21	NM_004168.2
98	1021	Genitourinary tract	1910105767	3.85	BRAF	CN_Amp	NA	8.97	NM_004333.4
99	1021	Genitourinary tract	2002060463	3.85	POLE	Nonsense_Mutation	p.R1371*	18.00	NM_006231.2
99	1021	Genitourinary tract	2002060463	3.85	HIST1H3B	Missense_Mutation	p.A96V	5.50	NM_003537.3
99	1021	Genitourinary tract	2002060463	3.85	APC	Nonsense_Mutation	p.E1309*	2.00	NM_000038.5
99	1021	Genitourinary tract	2002060463	3.85	APC	Nonsense_Mutation	p.C995*	1.80	NM_000038.5

100	1021	Genitourinary tract	1908304345	0.96	SF3B1	Missense_Mutation	p.R625L	5.50	NM_012433.2
101	1021	Genitourinary tract	2103025640	0.00	CCNE1	CN_Amp	NA	5.00	NM_001238.2
101	1021	Genitourinary tract	2103025640	0.00	CIC	CN_Del	NA	0.20	NM_015125.3
102	1021	Genitourinary tract	2108103095-1	1.92	TP53	Missense_Mutation	p.R273H	82.90	NM_000546.5
102	1021	Genitourinary tract	2108103095-1	1.92	DDR1	Missense_Mutation	p.V789M	29.30	NM_001954.4
102	1021	Genitourinary tract	2108103095-1	1.92	FOXP1	CN_Amp	NA	5.80	NM_001244814.1
102	1021	Genitourinary tract	2108103095-1	1.92	MLH3	CN_Amp	NA	4.40	NM_001040108.1
102	1021	Genitourinary tract	2108103095-1	1.92	PARP1	CN_Amp	NA	4.20	NM_001618.3
102	1021	Genitourinary tract	2108103095-1	1.92	CASP8	CN_Amp	NA	3.80	NM_001080125.1
102	1021	Genitourinary tract	2108103095-1	1.92	TBX3	CN_Amp	NA	0.40	NM_016569.3
102	1021	Genitourinary tract	2108103095-1	1.92	MET	CN_Amp	NA	0.20	NM_000245.2
102	1021	Genitourinary tract	2108103095-1	1.92	MEF2B-TLE2	Fusion	NA	28.80	NM_001145785.1; NM_003260.4
103	1021	Genitourinary tract	2203164172-1	1.92	NRAS	Missense_Mutation	p.Q61H	31.40	NM_002524.4
103	1021	Genitourinary tract	2203164172-1	1.92	SMARCA4	Missense_Mutation	p.I1173V	16.70	NM_003072.3
103	1021	Genitourinary tract	2203164172-1	1.92	MDM4	CN_Amp	NA	5.60	NM_002393.4
103	1021	Genitourinary tract	2203164172-1	1.92	IKBKE	CN_Amp	NA	5.00	NM_014002.3
103	1021	Genitourinary tract	2203164172-1	1.92	EXT1	CN_Amp	NA	5.00	NM_000127.2
103	1021	Genitourinary tract	2203164172-1	1.92	MYC	CN_Amp	NA	3.80	NM_002467.4
103	1021	Genitourinary tract	2203164172-1	1.92	ADAM18-FGFR1	Fusion	NA	43.00	NM_014237.2;NM _023110.2
104	1021	Head and neck	2110115918-1	4.80	ASXL2	Nonsense_Mutation	p.R302*	77.90	NM_018263.4
104	1021	Head and neck	2110115918-1	4.80	KIT	Missense_Mutation	p.K642E	77.20	NM_000222.2
104	1021	Head and neck	2110115918-1	4.80	ADCY1	Missense_Mutation	p.P1103L	44.80	NM_021116.2
104	1021	Head and neck	2110115918-1	4.80	SF3B1	Missense_Mutation	p.R625L	41.20	NM_012433.2
104	1021	Head and neck	2110115918-1	4.80	MLH3	Missense_Mutation	p.H265R	38.60	NM_001040108.1
104	1021	Head and neck	2110115918-1	4.80	KIT	CN_Amp	NA	5.40	NM_000222.2
105	1021	Uvea	2106070318-1	3.84	GNA11	Missense_Mutation	p.Q209L	31.60	NM_002067.2
105	1021	Uvea	2106070318-1	3.84	SF3B1	Missense_Mutation	p.R625H	18.30	NM_012433.2
105	1021	Uvea	2106070318-1	3.84	RAB35	Missense_Mutation	p.K137R	33.10	NM_006861.6
105	1021	Uvea	2106070318-1	3.84	MLL2	Missense_Mutation	p.R2282W	32.40	NM_003482.3
106	1021	Uvea	2107202008-1	1.92	SF3B1	Missense_Mutation	p.R625L	15.10	NM_012433.2
106	1021	Uvea	2107202008-1	1.92	GNAQ	Missense_Mutation	p.Q209P	9.10	NM_002072.3
106	1021	Uvea	2107202008-1	1.92	DAXX	CN_Amp	NA	4.60	NM_001141970.1
106	1021	Uvea	2107202008-1	1.92	MYC	CN_Amp	NA	4.20	NM_002467.4
107	1021	Head and neck	2106180719-1	2.88	NF1	Frame_Shift	p.V723Gfs*26	70.00	NM_000267.3
107	1021	Head and neck	2106180719-1	2.88	GLI3	Missense_Mutation	p.A762V	50.40	NM_000168.5
107	1021	Head and neck	2106180719-1	2.88	MITF	Frame_Shift	p.E208Rfs*18	19.80	NM_198159.2
107	1021	Head and neck	2106180719-1	2.88	KRAS	CN_Amp	NA	23.40	NM_033360.2

107	1021	Head and neck	2106180719-1	2.88	RAD51C	CN_Amp	NA	17.20	NM_058216.1
107	1021	Head and neck	2106180719-1	2.88	CDK4	CN_Amp	NA	15.40	NM_000075.3
107	1021	Head and neck	2106180719-1	2.88	RNF43	CN_Amp	NA	10.40	NM_017763.4
107	1021	Head and neck	2106180719-1	2.88	MDM2	CN_Amp	NA	8.40	NM_002392.4
107	1021	Head and neck	2106180719-1	2.88	ERBB3	CN_Amp	NA	6.60	NM_001982.3
107	1021	Head and neck	2106180719-1	2.88	IL7R	CN_Amp	NA	5.20	NM_002185.3
107	1021	Head and neck	2106180719-1	2.88	BRAF	CN_Amp	NA	4.80	NM_004333.4
107	1021	Head and neck	2106180719-1	2.88	TBX3	CN_Amp	NA	4.20	NM_016569.3
107	1021	Head and neck	2106180719-1	2.88	DOT1L	CN_Amp	NA	4.00	NM_032482.2
107	1021	Head and neck	2106180719-1	2.88	RNF43-IFLTD1	Fusion	NA	36.10	NM_017763.4;NM _001145727.2
108	1021	Anorectum	2108123220-1	1.92	SF3B1	Missense_Mutation	p.R625C	35.80	NM_012433.2
108	1021	Anorectum	2108123220-1	1.92	ERBB4	Missense_Mutation	p.R847C	34.80	NM_005235.2
108	1021	Anorectum	2108123220-1	1.92	FANCG	CN_Amp	NA	4.80	NM_004629.1
108	1021	Anorectum	2108123220-1	1.92	PAX5	CN_Amp	NA	4.60	NM_016734.1
108	1021	Anorectum	2108123220-1	1.92	RAC1	CN_Amp	NA	4.40	NM_018890.3
108	1021	Anorectum	2108123220-1	1.92	PMS2	CN_Amp	NA	4.40	NM_000535.5
108	1021	Anorectum	2108123220-1	1.92	VEGFA	CN_Amp	NA	4.40	NM_001171623.1
108	1021	Anorectum	2108123220-1	1.92	CCNE1	CN_Amp	NA	4.20	NM_001238.2
108	1021	Anorectum	2108123220-1	1.92	RPTOR	CN_Amp	NA	4.20	NM_020761.2
109	1021	Anorectum	2103126142	2.88	PTEN	Missense_Mutation	p.R15S	64.00	NM_000314.4
109	1021	Anorectum	2103126142	2.88	TERT	5'UTR	c.-124C>T	0.41	NM_198253.2
109	1021	Anorectum	2103126142	2.88	FLT4	Missense_Mutation	p.R1135S	0.27	NM_182925.4
109	1021	Anorectum	2103126142	2.88	PIK3R1	In_Frame	c.1300-14_1315delTTTAATCTTTCTAGGATCAAGTTGTCAAAGinsCTTCT	0.06	NM_181523.2
109	1021	Anorectum	2103126142	2.88	CDKN2A	CN_Del	NA	0.60	NM_000077.4
109	1021	Anorectum	2103126142	2.88	CDKN2B	CN_Del	NA	0.40	NM_004936.3
110	1021	Anorectum	2108243791-1	0.96	PTEN	Missense_Mutation	p.H93Q	77.40	NM_000314.4
110	1021	Anorectum	2108243791-1	0.96	PDGFRA	CN_Amp	NA	4.40	NM_006206.4
110	1021	Anorectum	2108243791-1	0.96	MCL1	CN_Amp	NA	4.20	NM_021960.4
110	1021	Anorectum	2108243791-1	0.96	KIT	CN_Amp	NA	4.20	NM_000222.2
110	1021	Anorectum	2108243791	0.96	KDR	CN_Amp	NA	4.20	NM_002253.2
110	1021	Anorectum	2108243791-1	0.96	RECQL4	CN_Amp	NA	4.00	NM_004260.3
110	1021	Anorectum	2108243791-1	0.96	FGFR1	CN_Amp	NA	3.80	NM_023110.2
110	1021	Anorectum	2108243791-1	0.96	SDHA	CN_Amp	NA	3.80	NM_004168.2
110	1021	Anorectum	2108243791-1	0.96	CIC	CN_Amp	NA	0.20	NM_015125.3
111	1021	Uvea	2109165009	3.84	BAP1	Missense_Mutation	p.F168S	56.40	NM_004656.2

111	1021	Uvea	2109165009	3.84	GNAQ	Missense_Mutation	p.Q209P	41.90	NM_002072.3
111	1021	Uvea	2109165009	3.84	FUBP1	Missense_Mutation	p.P351R	35.30	NM_003902.3
111	1021	Uvea	2109165009	3.84	RPTOR	Missense_Mutation	p.D1044Y	29.00	NM_020761.2
111	1021	Uvea	2109165009	3.84	MYC	CN_Amp	NA	4.00	NM_002467.4
111	1021	Uvea	2109165009	3.84	RECQL4	CN_Amp	NA	4.20	NM_004260.3
111	1021	Uvea	2109165009	3.84	NBN	CN_Amp	NA	4.20	NM_002485.4
111	1021	Uvea	2109165009	3.84	EXT1	CN_Amp	NA	4.00	NM_000127.2
112	1021	Uvea	2110216566	2.88	GNAQ	Missense_Mutation	p.G48L	53.60	NM_002072.3
112	1021	Uvea	2110216566	2.88	SF3B1	Missense_Mutation	p.D781N	51.70	NM_012433.2
112	1021	Uvea	2110216566	2.88	SF3B1	Missense_Mutation	p.N1218S	13.30	NM_012433.2
113	1021	Uvea	2201171356	2.88	BAP1	Splice_Site	c.437+2T>A	12.80	NM_004656.2
113	1021	Uvea	2201171356	2.88	GNA11	Missense_Mutation	p.Q209L	7.20	NM_002067.2
113	1021	Uvea	2201171356	2.88	MYC	CN_Amp	NA	4.00	NM_002467.4
114	1021	Anorectum	2203154119	68.16	TP53	Missense_Mutation	p.C141Y	64.50	NM_000546.5
114	1021	Anorectum	2203154119	68.16	TAF1	Missense_Mutation	p.S460P	33.90	NM_004606.3
114	1021	Anorectum	2203154119	68.16	ERBB4	Missense_Mutation	p.A455T	30.50	NM_005235.2
114	1021	Anorectum	2203154119	68.16	MSH6	Nonsense_Mutation	p.Q939*	27.40	NM_000179.2
114	1021	Anorectum	2203154119	68.16	ERCC3	Missense_Mutation	p.R553K	26.90	NM_000122.1
114	1021	Anorectum	2203154119	68.16	PPP2R1A	Missense_Mutation	p.V306I	26.50	NM_014225.5
114	1021	Anorectum	2203154119	68.16	FANCA	Missense_Mutation	p.I1081V	24.10	NM_000135.2
114	1021	Anorectum	2203154119	68.16	FLT1	Missense_Mutation	p.T419I	23.30	NM_002019.4
114	1021	Anorectum	2203154119	68.16	CLDN18	Missense_Mutation	p.G17R	21.70	NM_016369.3
114	1021	Anorectum	2203154119	68.16	CDH11	Missense_Mutation	p.A396T	20.50	NM_001797.2
114	1021	Anorectum	2203154119	68.16	EPHB1	Missense_Mutation	p.S766F	18.20	NM_004441.4
114	1021	Anorectum	2203154119	68.16	NTRK2	Missense_Mutation	p.S414N	18.20	NM_006180.3
114	1021	Anorectum	2203154119	68.16	RBM10	Missense_Mutation	p.A884T	17.70	NM_001204468.1
114	1021	Anorectum	2203154119	68.16	PALB2	Missense_Mutation	p.L206F	16.30	NM_024675.3
114	1021	Anorectum	2203154119	68.16	POM121L12	Missense_Mutation	p.R208W	15.90	NM_182595.3
114	1021	Anorectum	2203154119	68.16	SLC22A18	Missense_Mutation	p.S262F	15.70	NM_183233.2
114	1021	Anorectum	2203154119	68.16	MLL2	Missense_Mutation	p.G4603D	15.70	NM_003482.3
114	1021	Anorectum	2203154119	68.16	BRD4	Missense_Mutation	p.P777S	15.60	NM_058243.2
114	1021	Anorectum	2203154119	68.16	PTCH1	Missense_Mutation	p.P1210S	15.00	NM_000264.3
114	1021	Anorectum	2203154119	68.16	CDKN2B	Missense_Mutation	p.L67F	14.90	NM_004936.3
114	1021	Anorectum	2203154119	68.16	MET	Missense_Mutation	p.G1085R	14.70	NM_000245.2
114	1021	Anorectum	2203154119	68.16	RPS6KB2	Missense_Mutation	p.W339*	14.50	NM_003952.2
114	1021	Anorectum	2203154119	68.16	NCOR1	Missense_Mutation	p.R671K	14.00	NM_006311.3
114	1021	Anorectum	2203154119	68.16	NOTCH1	Missense_Mutation	p.A1740T	13.90	NM_017617.3
114	1021	Anorectum	2203154119	68.16	RPTOR	Missense_Mutation	p.S719F	13.80	NM_020761.2
114	1021	Anorectum	2203154119	68.16	AXIN2	Nonsense_Mutation	p.W111*	13.00	NM_004655.3

114	1021	Anorectum	2203154119	68.16	RNF43	Missense_Mutation	p.A623V	12.80	NM_017763.4
114	1021	Anorectum	2203154119	68.16	RAD54L	Missense_Mutation	p.T169I	12.40	NM_003579.3
114	1021	Anorectum	2203154119	68.16	PTGS1	Missense_Mutation	p.G532S	12.20	NM_000962.3
114	1021	Anorectum	2203154119	68.16	FOXP1	Missense_Mutation	p.P626L	12.00	NM_001244814.1
114	1021	Anorectum	2203154119	68.16	EPHA5	Missense_Mutation	p.V475A	12.00	NM_004439.5
114	1021	Anorectum	2203154119	68.16	PARP1	Missense_Mutation	p.G723S	11.60	NM_001618.3
114	1021	Anorectum	2203154119	68.16	PTPRT	Missense_Mutation	p.P376L	11.60	NM_133170.3
114	1021	Anorectum	2203154119	68.16	ZFHX3	Missense_Mutation	p.E466K	11.20	NM_006885.3
114	1021	Anorectum	2203154119	68.16	SRC	Missense_Mutation	p.N535D	10.70	NM_198291.1
114	1021	Anorectum	2203154119	68.16	H3F3A	Missense_Mutation	p.G34E	10.50	NM_002107.4
114	1021	Anorectum	2203154119	68.16	TOP1	Missense_Mutation	p.A476V	10.50	NM_003286.2
114	1021	Anorectum	2203154119	68.16	APC	Missense_Mutation	p.V1452I	10.30	NM_000038.5
114	1021	Anorectum	2203154119	68.16	TRRAP	Missense_Mutation	p.S3649N	10.30	NM_001244580.1
114	1021	Anorectum	2203154119	68.16	NTRK3	Missense_Mutation	p.G473D	10.10	NM_001012338.2
114	1021	Anorectum	2203154119	68.16	NOTCH4	Missense_Mutation	p.L221F	10.00	NM_004557.3
114	1021	Anorectum	2203154119	68.16	JAK1	Splice_Site	c.3140+1G>A	9.90	NM_002227.2
114	1021	Anorectum	2203154119	68.16	EGFR	Missense_Mutation	p.S720F	9.90	NM_005228.3
114	1021	Anorectum	2203154119	68.16	NPM1	Missense_Mutation	p.G69S	9.00	NM_002520.6
114	1021	Anorectum	2203154119	68.16	CYP2D6	Missense_Mutation	p.V79I	8.90	NM_000106.5
114	1021	Anorectum	2203154119	68.16	PAX5	Missense_Mutation	p.S133N	8.80	NM_016734.1
114	1021	Anorectum	2203154119	68.16	POT1	Missense_Mutation	p.D577N	8.50	NM_015450.2
114	1021	Anorectum	2203154119	68.16	MLL3	Missense_Mutation	p.G4632S	8.40	NM_170606.2
114	1021	Anorectum	2203154119	68.16	GRM3	Missense_Mutation	p.C804Y	7.80	NM_000840.2
114	1021	Anorectum	2203154119	68.16	CDK6	Missense_Mutation	p.S296F	7.80	NM_001145306.1
114	1021	Anorectum	2203154119	68.16	FANCM	Nonsense_Mutation	p.Q1730*	7.60	NM_020937.2
114	1021	Anorectum	2203154119	68.16	FANCM	Missense_Mutation	p.T303I	6.80	NM_020937.2
114	1021	Anorectum	2203154119	68.16	VEGFA	Missense_Mutation	p.A30T	5.90	NM_001171623.1
114	1021	Anorectum	2203154119	68.16	FANCE	CN_Amp	NA	4.80	NM_021922.2
114	1021	Anorectum	2203154119	68.16	RAD52	CN_Amp	NA	4.40	NM_134424.2
114	1021	Anorectum	2203154119	68.16	BLM	CN_Amp	NA	4.00	NM_000057.2
114	1021	Anorectum	2203154119	68.16	IL7R	CN_Amp	NA	4.00	NM_002185.3
114	1021	Anorectum	2203154119	68.16	RAD50	CN_Amp	NA	3.80	NM_005732.3
115	1021	Head and neck	2001089763	3.84	MLH3	Missense_Mutation	p.S1297F	10.40	NM_001040108.1
115	1021	Head and neck	2001089763	3.84	FGFR3	Missense_Mutation	p.G40S	9.70	NM_000142.4
115	1021	Head and neck	2001089763	3.84	SHH	Missense_Mutation	p.D95N	6.40	NM_000193.2
115	1021	Head and neck	2001089763	3.84	ERBB3	Missense_Mutation	p.E928K	5.10	NM_001982.3
116	1021	Anorectum	1911077035	5.76	NF1	Missense_Mutation	p.G629R	14.90	NM_000267.3
116	1021	Anorectum	1911077035	5.76	KIT	Missense_Mutation	p.I798V	79.10	NM_000222.2
116	1021	Anorectum	1911077035	5.76	STAT3	Missense_Mutation	p.S476Y	15.90	NM_139276.2

116	1021	Anorectum	1911077035	5.76	MET	Missense_Mutation	p.H1088Q	13.10	NM_000245.2
116	1021	Anorectum	1911077035	5.76	CTNNB1	Frame_Shift	p.V589Yfs*19	9.00	NM_001904.3
116	1021	Anorectum	1911077035	5.76	GAB2	Missense_Mutation	p.E362Q	3.40	NM_080491.2
116	1021	Anorectum	1911077035	5.76	SLX4	Missense_Mutation	p.R1062C	2.90	NM_032444.2
116	1021	Anorectum	1911077035	5.76	EPHA3	Missense_Mutation	p.M155I	2.40	NM_005233.5
116	1021	Anorectum	1911077035	5.76	MDM2	CN_Amp	NA	9.20	NM_002392.4
116	1021	Anorectum	1911077035	5.76	CCND1	CN_Amp	NA	31.80	NM_053056.2
116	1021	Anorectum	1911077035	5.76	FGF19	CN_Amp	NA	25.00	NM_005117.2
116	1021	Anorectum	1911077035	5.76	FGF4	CN_Amp	NA	22.80	NM_002007.2
116	1021	Anorectum	1911077035	5.76	FGF3	CN_Amp	NA	23.20	NM_005247.2
116	1021	Anorectum	1911077035	5.76	KRAS	CN_Amp	NA	5.60	NM_033360.2
116	1021	Anorectum	1911077035	5.76	CDK4	CN_Amp	NA	12.00	NM_000075.3
116	1021	Anorectum	1911077035	5.76	IFNG	CN_Amp	NA	10.00	NM_000619.2
116	1021	Anorectum	1911077035	5.76	PMS1	CN_Amp	NA	5.60	NM_000534.4
116	1021	Anorectum	1911077035	5.76	SF3B1	CN_Amp	NA	9.20	NM_012433.2
116	1021	Anorectum	1911077035	5.76	MAPK1	CN_Amp	NA	6.00	NM_002745.4
116	1021	Anorectum	1911077035	5.76	KIT	CN_Amp	NA	5.60	NM_000222.2
116	1021	Anorectum	1911077035	5.76	KDR	CN_Amp	NA	5.40	NM_002253.2
116	1021	Anorectum	1911077035	5.76	ATM	Splice_Site	c.331+5G>A	NA	NM_000051.3
