# Supplementary material for: Clinical significance of genetic profiling based on different anatomic sites in patients with mucosal melanoma who received or did not receive immune checkpoint inhibitors
Source: Cancer Cell Int. 2023 Aug 30;23:187. doi: 10.1186/s12935-023-03032-3 (PMC10469937; doi:10.1186/s12935-023-03032-3)
Supplement: Supplementary file 3 — Supplementary Material 3 [file 12935_2023_3032_MOESM3_ESM.pdf]

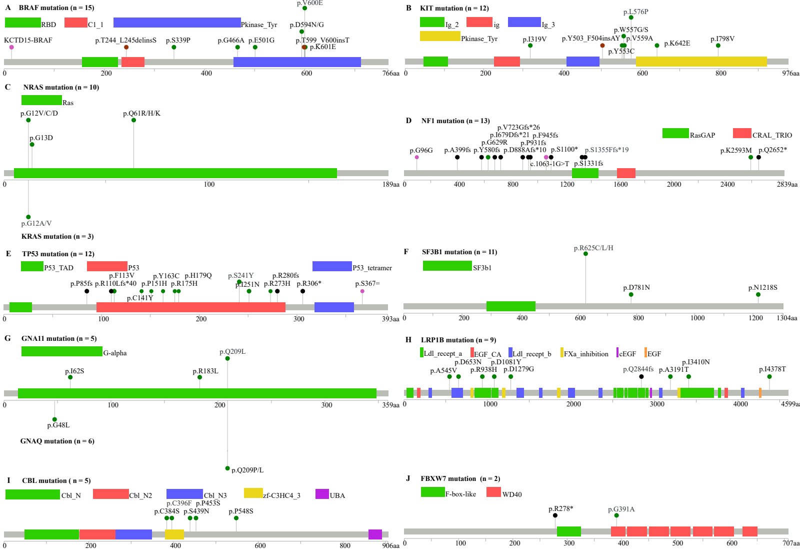

Supplementary Figure S1. Positions of BRAF, KIT, RAS, NF1, TP53, SF3B1, GNA11, GNAQ, LRP1B, CBL and FBXW7 somatic mutations in the protein.
