# Supplementary figures and images for: Clinical significance of genetic profiling based on different anatomic sites in patients with mucosal melanoma who received or did not receive immune checkpoint inhibitors
Source: Cancer Cell Int. 2023 Aug 30;23:187. doi: 10.1186/s12935-023-03032-3 (PMC10469937; doi:10.1186/s12935-023-03032-3)

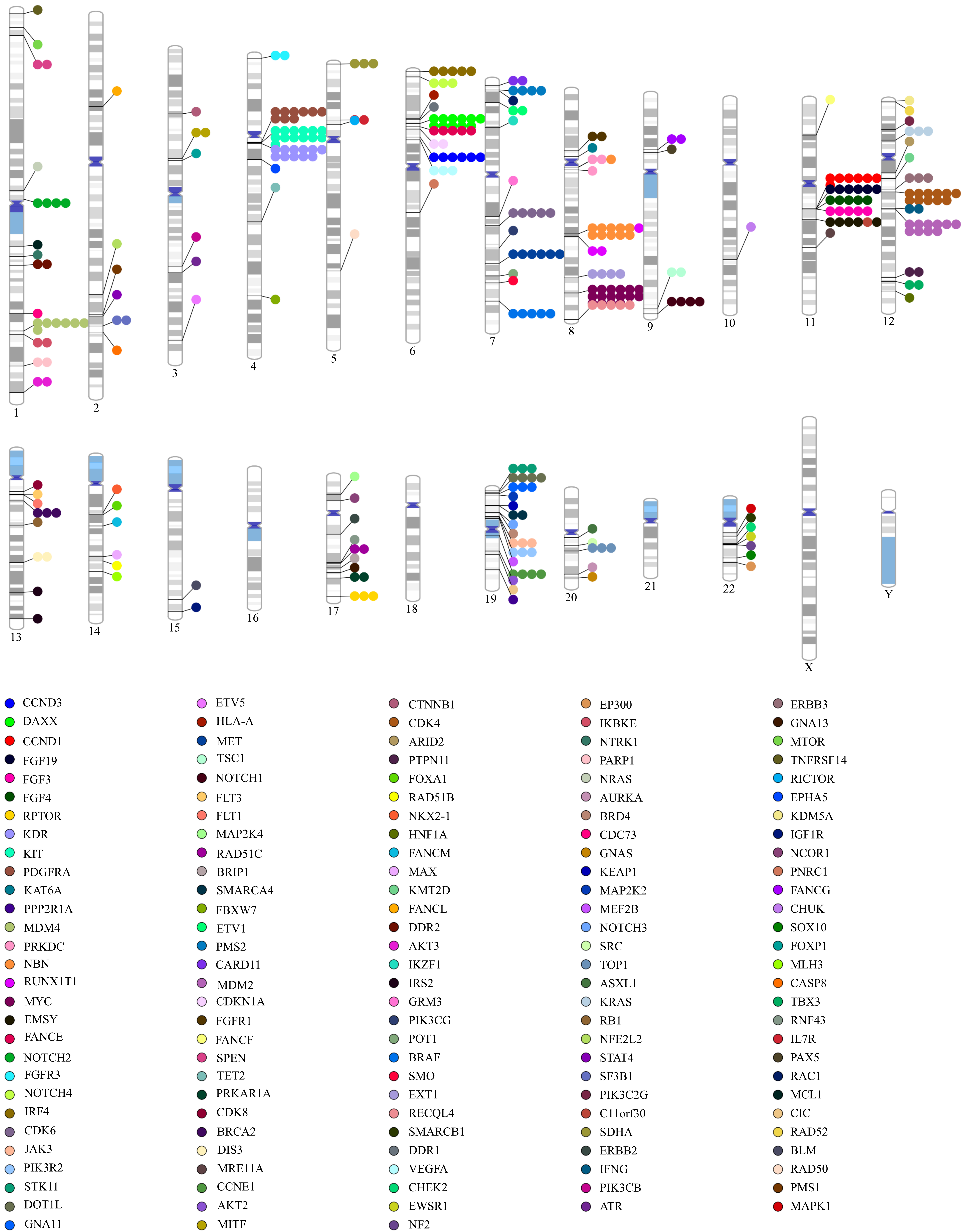

**Supplementary Figure S2.** The chromosomal distribution of recurrently copy number variants.

Supplement: Supplementary file 4 — Supplementary Material 4 [file 12935_2023_3032_MOESM4_ESM.pdf]
