# Supplementary material for: Clinical significance of genetic profiling based on different anatomic sites in patients with mucosal melanoma who received or did not receive immune checkpoint inhibitors
Source: Cancer Cell Int. 2023 Aug 30;23:187. doi: 10.1186/s12935-023-03032-3 (PMC10469937; doi:10.1186/s12935-023-03032-3)
Supplement: Supplementary file 5 — Supplementary Material 5 [file 12935_2023_3032_MOESM5_ESM.pdf]

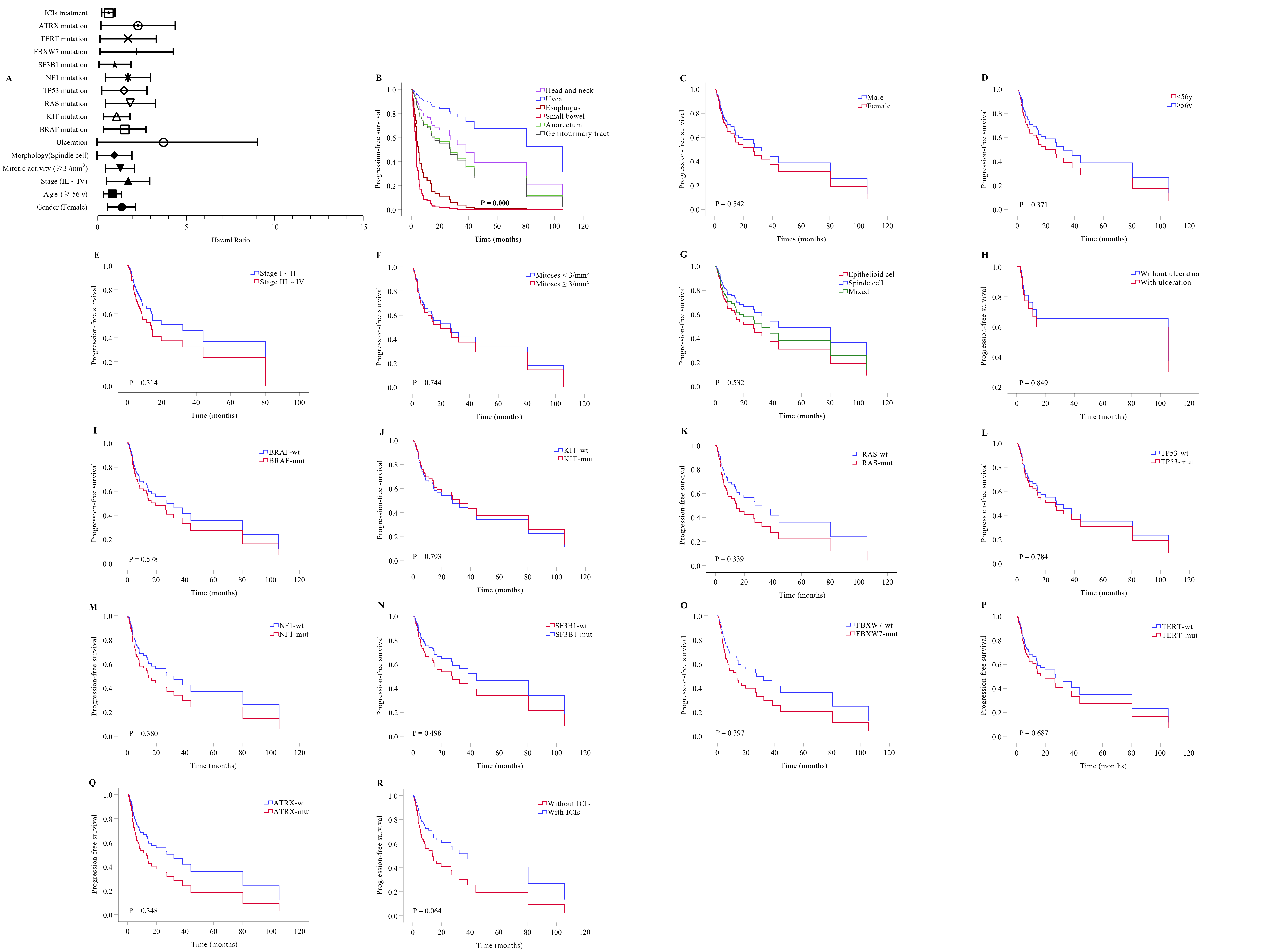

Supplementary Figure S3. Kaplan–Meier survival analysis in MM. Forest plot and Kaplan–Meier survival curves about PFS were performed in the patients with different clinicopathological features and significantly mutated genes.

Abbreviation: MM, mucosal melanoma; PFS, progression-free survival.
